# Translatome profiling reveals *Itih4* as a novel smooth muscle cell–specific gene in atherosclerosis

**DOI:** 10.1093/cvr/cvae028

**Published:** 2024-01-30

**Authors:** Aarthi Ravindran, Lari Holappa, Henri Niskanen, Ilya Skovorodkin, Susanna Kaisto, Mustafa Beter, Miika Kiema, Ilakya Selvarajan, Valtteri Nurminen, Einari Aavik, Rédouane Aherrahrou, Sanna Pasonen-Seppänen, Vittorio Fortino, Johanna P Laakkonen, Seppo Ylä-Herttuala, Seppo Vainio, Tiit Örd, Minna U Kaikkonen

**Affiliations:** A.I.Virtanen Institute for Molecular Sciences, University of Eastern Finland, Neulaniementie 2, 70211 Kuopio, Finland; A.I.Virtanen Institute for Molecular Sciences, University of Eastern Finland, Neulaniementie 2, 70211 Kuopio, Finland; A.I.Virtanen Institute for Molecular Sciences, University of Eastern Finland, Neulaniementie 2, 70211 Kuopio, Finland; Disease networks research unit, Faculty of Biochemistry and Molecular Medicine, Kvantum Institute, Infotech Oulu, University of Oulu, Oulu, Finland; Disease networks research unit, Faculty of Biochemistry and Molecular Medicine, Kvantum Institute, Infotech Oulu, University of Oulu, Oulu, Finland; A.I.Virtanen Institute for Molecular Sciences, University of Eastern Finland, Neulaniementie 2, 70211 Kuopio, Finland; A.I.Virtanen Institute for Molecular Sciences, University of Eastern Finland, Neulaniementie 2, 70211 Kuopio, Finland; A.I.Virtanen Institute for Molecular Sciences, University of Eastern Finland, Neulaniementie 2, 70211 Kuopio, Finland; A.I.Virtanen Institute for Molecular Sciences, University of Eastern Finland, Neulaniementie 2, 70211 Kuopio, Finland; A.I.Virtanen Institute for Molecular Sciences, University of Eastern Finland, Neulaniementie 2, 70211 Kuopio, Finland; A.I.Virtanen Institute for Molecular Sciences, University of Eastern Finland, Neulaniementie 2, 70211 Kuopio, Finland; Institute for Cardiogenetics, Universität zu Lübeck, 23562 Lübeck, Germany; DZHK (German Centre for Cardiovascular Research), Partner Site Hamburg/Kiel/Lübeck, University Heart Centre Lübeck, 23562 Lübeck, Germany; Institute of Biomedicine, School of Medicine, Faculty of Health Sciences, University of Eastern Finland, Kuopio, Finland; Institute of Biomedicine, School of Medicine, Faculty of Health Sciences, University of Eastern Finland, Kuopio, Finland; A.I.Virtanen Institute for Molecular Sciences, University of Eastern Finland, Neulaniementie 2, 70211 Kuopio, Finland; A.I.Virtanen Institute for Molecular Sciences, University of Eastern Finland, Neulaniementie 2, 70211 Kuopio, Finland; Disease networks research unit, Faculty of Biochemistry and Molecular Medicine, Kvantum Institute, Infotech Oulu, University of Oulu, Oulu, Finland; A.I.Virtanen Institute for Molecular Sciences, University of Eastern Finland, Neulaniementie 2, 70211 Kuopio, Finland; A.I.Virtanen Institute for Molecular Sciences, University of Eastern Finland, Neulaniementie 2, 70211 Kuopio, Finland

**Keywords:** Smooth muscle cells, Translatome, TRAP-seq, Mouse models, Atherosclerosis, Gene regulation, *Itih4*, RPL10a

## Abstract

**Aims:**

Vascular smooth muscle cells (SMCs) and their derivatives are key contributors to the development of atherosclerosis. However, studying changes in SMC gene expression in heterogeneous vascular tissues is challenging due to the technical limitations and high cost associated with current approaches. In this paper, we apply translating ribosome affinity purification sequencing to profile SMC-specific gene expression directly from tissue.

**Methods and results:**

To facilitate SMC-specific translatome analysis, we generated SMC^TRAP^ mice, a transgenic mouse line expressing enhanced green fluorescent protein (EGFP)-tagged ribosomal protein L10a (EGFP-L10a) under the control of the SMC-specific αSMA promoter. These mice were further crossed with the atherosclerosis model Ldlr^−/−^, ApoB^100/100^ to generate SMC^TRAP−AS^ mice and used to profile atherosclerosis-associated SMCs in thoracic aorta samples of 15-month-old SMC^TRAP^ and SMC^TRAP-AS^ mice. Our analysis of SMC^TRAP-AS^ mice showed that EGFP-L10a expression was localized to SMCs in various tissues, including the aortic wall and plaque. The TRAP fraction demonstrated high enrichment of known SMC-specific genes, confirming the specificity of our approach. We identified several genes, including *Cemip*, *Lum*, *Mfge8*, *Spp1*, and *Serpina3*, which are known to be involved in atherosclerosis-induced gene expression. Moreover, we identified several novel genes not previously linked to SMCs in atherosclerosis, such as *Anxa4*, *Cd276*, inter-alpha-trypsin inhibitor-4 (*Itih4*), *Myof*, *Pcdh11x*, *Rab31*, *Serpinb6b*, *Slc35e4*, *Slc8a3*, and *Spink5*. Among them, we confirmed the SMC-specific expression of *Itih4* in atherosclerotic lesions using immunofluorescence staining of mouse aortic roots and spatial transcriptomics of human carotid arteries. Furthermore, our more detailed analysis of *Itih4* showed its link to coronary artery disease through the colocalization of genome-wide association studies, splice quantitative trait loci (QTL), and protein QTL signals.

**Conclusion:**

We generated a SMC-specific TRAP mouse line to study atherosclerosis and identified *Itih4* as a novel SMC-expressed gene in atherosclerotic plaques, warranting further investigation of its putative function in extracellular matrix stability and genetic evidence of causality.

**Time of primary review: 24 days**

**See the editorial comment for this article ‘NOT lost in translation: translatome mapping as a novel approach to identify regulators of atherosclerosis’, by L. Matic *et al*., https://doi.org/10.1093/cvr/cvae089.**

## 1. Introduction

Cardiovascular diseases are a major reason for the death worldwide, and vascular smooth muscle cells (SMCs) are the primary structural cells of blood vessels. SMCs maintain the elasticity and strength of blood vessels by synthesis and organization of extracellular matrix (ECM).^1^ SMC function is closely linked with other cell types, such as endothelial cells (ECs), for the relaxation and contraction of blood vessels, and with adventitia fibroblasts for producing collagen fibres to form the peripheral structure to preserve the vascular integrity at high pressures. SMCs are typically differentiated (expressing contractile markers) and quiescent in healthy arteries, but in pathological conditions, they can undergo dedifferentiation, becoming more proliferative, migratory, and capable of synthesizing excessive ECM. This shift to a ‘synthetic’ phenotype where the SMCs undergo broad spectrum of structural and functional changes has been observed in various cardiovascular diseases.^2,3^

Recent advances in cell lineage tracing and single-cell RNA sequencing (scRNA-Seq) have revealed the heterogeneity of SMCs in atherosclerosis. These studies have suggested that a subset of contractile SMCs can undergo a transition to a multipotent state, capable of adopting either chondro-osteogenic, inflammatory, or ECM-rich cell states.^4–7^ However, scRNA-Seq techniques also have limitations, including high cost, low sample size, the potential for tissue dissociation artefacts,^8–10^ and the inability to detect lowly expressed genes.^11–13^ Furthermore, many commonly used platforms for scRNA-Seq studies that rely on 3′ tag-based sequencing has a higher likelihood of missing longer transcripts, which can result in fewer differentially expressed genes compared with whole transcript sequencing.^14–16^

In this study, we adapted the translating ribosome affinity purification (TRAP) technique^17–19^ to selectively isolate polysomes (and their associated mRNA) from SMCs in atherosclerotic (Ldlr^−/−^, ApoB^100/100^) mice. Our results demonstrate the specificity and sensitivity of our approach in detecting genes previously not linked to SMCs in atherosclerosis.

## 2. Methods

### 2.1 Mouse line generation

The αSMA-enhanced green fluorescent protein (EGFP)-L10a (SMC^TRAP^) mice line was developed by first cloning the mouse αSMA promoter into the pGL4.10 vector (Promega) between the KpnI and XhoI restriction sites. The promoter fragment was polymerase chain reaction (PCR)-amplified from genomic DNA using the following primers: forward (5′-AAAA GGTACC ACGCGT ACACCATAAAACAAGTGCATGAGCC-3′ with KpnI and Mlu I) and reverse (5′-AAAA CTCGAG GGGCCC TAGCTGGAGCAGCGTCTCAGG-3′ with XhoI and ApaI). The αSMA promoter from the pGL4.10 vector was then inserted into the Cfms-EGFP-L10a transgenic construct by replacing the insert (*Csf1r* promoter and intron 2) using MluI and ApaI digestion.^20^ Transgenic mice (SMC^TRAP^) were generated by microinjecting the purified and dialyzed transgenic construct into oocytes. These mice were subsequently crossed with atherosclerosis-prone mice (Ldlr^−/−^, ApoB^100/100^) from the Jackson Laboratory (strain no. 003000)^21^ to create SMC^TRAP-AS^ mice. The EGFP-L10a transgene was genotyped using PCR (primers 5′-CCTACGGCGTGCAGTGCTTCA-3′ and 5′-CGGCGAGCTGCACGCTGCCGT-3′) that generates a 345 bp product, while the mutated *Ldlr* and *ApoB* alleles were genotyped using protocols provided by the Jackson Laboratory.^21^

### 2.2 Animal experiments

The mice were maintained according to guidelines set by the Ethical Committee of University of Eastern Finland and the National Experiment Animal Board of Finland, in accordance with the Finnish Act on Animal Experimentation and directive 2010/63/EU of the European Parliament and European Council. The list of mouse strains utilized in this study can be found in Supplementary material online, *Table S1*. These mice were housed under standard conditions with a 12 h light/dark cycle and were given access to a rodent chow diet (Teklad Global Rodent diet 2016 for maintenance and Teklad Global Rodent 2018s for breeding) and water *ad libitum*.

At the age of 3 months, two groups of mice were established for plaque phenotype and serum biochemistry studies: SMC^TRAP-AS^ mice and a control group lacking the TRAP construct (Ldlr^−/−^ ApoB^100/100^, Athero). The number of mice used for each analysis is stated in the figure legends. Both groups were fed a Western-style high-fat diet (HFD) (Teklad TD.88137) *ad libitum* for 4 months. For translating ribosome affinity purification sequencing (TRAP-Seq), aortas were collected from 15-month-old SMC^TRAP-AS^ mice (*n* = 9, 3 males and 6 females) and SMC^TRAP^ mice (*n* = 9, 3 males and 6 females) that had been fed with chow diet (Teklad Global Rodent diet 2016). For immunofluorescence imaging of EGFP expression, we used 12-month-old female SMC^TRAP-AS^ mice that had been fed a HFD (Teklad TD.88137) for 10 weeks. For αSMA and EGFP, costaining in the aorta is done from mice (*n* = 3) fed with chow diet and aged 15 months old.

### 2.3 Tissue collection and processing

Mice were euthanized in CO_2_ chamber (Metos). The blood was collected to an ethylenediaminetetraacetic acid (EDTA) tube (BD Vacutainer) via cardiac puncture for plasma separation, and the remaining tissues were dissected while the mice were kept on ice after perfusing with ice-cold 1× phosphate buffered saline (PBS) (Gibco). The aorta was snap-frozen using liquid nitrogen for TRAP-Seq. Tissues used for immunohistology or immunofluorescence (aortic root, brachiocephalic artery) were fixed in 4% paraformaldehyde (PFA) (Sigma) for overnight at 4°C. After the fixation period, the tissue was transferred to a 15% sucrose (Merck) solution until they sank to the bottom. For paraffin embedding, the tissues were first processed in a Citadel 2000 (Thermo Fisher Scientific) for 19 h using several dehydration steps with xylene, ethanol, and paraffin. The processed tissue was then embedded in paraffin using a HistoCenter2 tissue embedding machine (Shadon) and stored at room temperature. Tissues collected for immunofluorescence analysis (aorta, liver, kidney, small intestine) were placed in optimal cutting temperature (OCT) compound (Tissue Tek) and stored at −70°C. Frozen tissues in OCT were sectioned using a Leica CM1950 cryostat or from paraffin blocks using a Thermo Scientific Microm-Cool-Cut HM 350 microtome, and the resulting sections of 5 µm were collected onto Superfrost microscopic slides (J1800AMNZ, Thermo Scientific).

### 2.4 Histological and immunofluorescence staining

The frozen tissues in OCT from SMA^TRAP-AS^ samples sections were imaged for EGFP expression using fluorescence microscopy, Nikon ECLIPSE Ni-E.

The haematoxylin and eosin (H&E) staining was used to investigate the development of plaque in the brachiocephalic artery between SMC^TRAP-AS^ and Ldlr^−/−^, ApoB^100/100^ (Athero) mice. The tissue sections were deparaffinized and rehydrated using a series of solvent washes (xylene, ethanol, and H_2_O) and stained with Delafield’s H&E staining and imaged using a Nikon ECLIPSE Ni-E microscope with a ×4 objective. The measurements of plaque were done in a blinded manner for all the samples using the microscope ROI software. Specifically, the perimeter of lumen, plaque, and total area of each brachiocephalic artery section were measured. The percentage of plaque area was calculated by dividing the perimeter-derived lumen area with plaque area.

To determine the expression of inter-alpha-trypsin inhibitor-4 (*Itih4*) gene and to further validate EGFP expression in cells, immunostainings were performed. The tissues were deparaffinized and rehydrated, and antigen retrieval was done using citrate buffer (pH 6.0) for 10 min in a pressure cooker. Thereafter, the sections were treated with 50 mM glycine for 20 min at room temperature to quench autofluorescence. The sections were blocked with 1% milk for 30 min at 37°C, followed by an overnight incubation at 4°C with the primary antibody against EGFP (1:100, rabbit monoclonal antibody 2956S, Cell Signaling Technology) and inter-alpha-trypsin inhibitor heavy chain 4 (ITIH4) (1:200 dilution, rabbit polyclonal antibody 24069-1-AP, Proteintech). After washing, the sections were incubated for 1 h, at room temperature with the secondary antibody (1:200, Fluorescein-goat-anti-rabbit, FI-1000, Vector Laboratories for ITIH4 and 1:200, Alexa Fluor 488-anti-rabbit, A11008, Invitrogen for EGFP). Nuclei were labelled with 4′,6-diamidino-2-phenylindole (DAPI) (1 μg/mL, Sigma-Aldrich). The sections were mounted with Vectashield (Vector H-1000, Vector Laboratories) mounting medium and imaged with a Zeiss LSM 700 confocal microscope using 405/488/555 nm diode lasers together with the appropriate emission filters (×20/0.5 PlanApo or ×40/1.3 NA oil objectives, Carl Zeiss AG, Jena, Germany). Mouse monoclonal anti-alpha smooth muscle actin Cy3 antibody (Sigma-Aldrich, C6198) was used to stain the SMCs.

### 2.5 Tissue pulverization and lysate preparation for TRAP-Seq

The frozen aortic tissue was pulverized using a TissueLyser II bead mill (QIAGEN) in the presence of a 5 mm stainless steel bead (QIAGEN REF-69989) at a frequency of 30 Hz for 2 min in a 2 mL round-bottom tube. To prevent thawing, TissueLyser II adapters and the sample were placed in dry ice along with the bead. The lysis buffer, consisting of a low salt buffer/homogenization buffer [50 mM Tris pH 7.4 (UltraPure, Fisher, 15567027), 100 mM KCl (Sigma, 60142), 12 mM MgCl_2_ (Invitrogen, AM9530G), and 1% IGEPAL CA630 (Sigma, I8896)] supplemented with 1 mM dithiothreitol (DTT) (Sigma, 10197777001), cOmplete Mini EDTA-free protease inhibitor (Roche 11836170001, one mini tablet/10 mL), 100 μg/mL cycloheximide (Sigma-Aldrich, 1810) prepared in dimethylsulfoxide (DMSO) (Sigma C1988-1G), and 0.2 U/μL murine RNase inhibitor (NEB M0314L) and 0.1 U/μL Superasin RNase inhibitor (Thermo Scientific AM2696), was prepared fresh and added to the pulverized tissue at a ratio of 1 mL per 100 mg tissue. The resulting mixture was pipetted to reduce viscosity and then centrifuged at 10 000 *g* for 10 min at 4°C to pellet debris.

### 2.6 Preparation of bead–protein L complex and sample preclearing for polysomal pulldown

Biotinylated Protein L (0.5 mg; Pierce #29997) was reconstituted in 100 µL of RNase-free water and added to 225 µL Dynabeads MyOne Streptavidin T1 (10 mg/mL; Invitrogen 65602). This bead–protein complex was used to capture the anti-GFP clone 19F7 and clone 19C8 antibodies (Antibody and Bioresource Core Facility, Memorial Sloan Kettering Cancer Center). Sample preclearing was done by washing the lysate once with Dynabeads MyOne Streptavidin T1 (10 mg/mL; Invitrogen 65602). After preclearing, 5% of the sample was separated and added to Qiagen buffer RLT (from the RNeasy Micro kit) for use in bulk RNA extraction (Input RNA).

### 2.7 Indirect target capture of polysomal mRNA tagged with EGFP using bead–protein L

The precleared lysate was incubated for 1–2 h at 4°C first with EGFP antibodies (10 µg of each 19C8 and 19F7 EGFP antibodies for every 100 mg of tissue), and subsequently, the bead–protein L complex (described above) was added to the lysate–antibody mixture and incubated for 1 h at 4°C. The beads bound to polysomal RNA were subsequently washed with a high-salt buffer to remove contaminants and improve the purity of the RNA extraction. This buffer contained 50 mM Tris (pH 7.4), 300 mM KCL, 12 mM MgCl_2_, 1% IGEPAL CA630 (Sigma), 1 mM DTT, and 100 µg/mL cycloheximide in DMSO (Sigma C1988-1G). To elute RNA from the beads, the Qiagen buffer RLT (from the RNeasy Micro kit) was added to the beads. This RNA sample forms the immunoprecipitated (IP) RNA.

### 2.8 RNA extraction and library preparation

For the RNA extraction and library preparation, thoracic aorta lysate in buffer RLT (IP and Input RNA) was homogenized using the QIAShredder instrument (QIAGEN, 79654). RNA was then isolated using the RNeasy Micro Plus kit (QIAGEN, 74034), and the quality and concentration of the isolated RNA were determined using the Qubit RNA HS Assay Kit (Invitrogen, Q32852) on a Qubit 2.0 Fluorometer (Invitrogen). The SMARTer Stranded Total RNA-Seq Kit v2—Pico Input Mammalian (TaKaRa, 634411, 634412) was used for library preparation, followed by single-end, 76 bp sequencing on an Illumina NextSeq 500 instrument.

### 2.9 RNA sequencing data processing and differential expression analysis

The sequencing data were processed using the Nfcore v1.4.2 RNA-Seq pipeline^22^ with the mm10 mouse reference genome and single-end, reverse stranded settings. As part of the pipeline, FastQC v0.11.8 was used and alignment was performed using the STAR 2.6.1 aligner. Differential expression (DE) analysis was performed using DEseq2^23^ to identify genes specifically expressed in SMCs (IP vs. Input) and in atherosclerosis (SMC^TRAP-AS^ vs. SMC^TRAP^). Prior to analysing male and female samples together, differences in gene expression between the sexes were investigated by dedicated DESeq2 analyses, as well as running TRAP-Seq analysis one sex at a time. The DE results comparing sexes as well as full TRAP-Seq results using male-only, female-only, or sexes combined are presented in Supplementary material online, *Tables 1–30*.

### 2.10 Pathway enrichment analysis

The Gene Ontology (GO) analysis of diverse gene sets related to atherosclerosis under SMC-enriched conditions (IP vs. IP) and total RNA (Input vs. Input) was conducted using gProfiler (31 January 2023).^24^ Additionally, we utilized the CNET plot feature in the enrichplot R package to depict the most enriched pathways associated with the 187 SMC-specific genes linked to atherosclerosis.

### 2.11 Blood plasma lipid profile

The EDTA plasma was obtained from blood via cardiac puncture and analysed for lipid content [HDL-C, LDL-C, triglyceride (TG), and total cholesterol (TC)] using an automated enzymatic method on a Konelab Prime 60i instrument (Thermo Scientific). Each measurement was performed with a minimum of 50 µL of plasma.

### 2.12 The comparison of SMC-enriched genes detected in TRAP-Seq and scRNA-Seq

Mouse aorta scRNA-Seq of the atherosclerosis mouse model matching our TRAP-Seq data (Ldlr^−/−^, ApoB^100/100^) was obtained from Örd *et al.*^25^ The 3-month HFD aorta scRNA-Seq was used to represent disease, and the wild-type chow diet aorta scRNA-Seq served as the non-diseased control. In scRNA-Seq analyses, SMC cells were compared with all non-SMC cells and DE was calculated using the Wilcoxon test. TRAP-Seq data were processed as described above. SMC-enriched genes were defined from TRAP-Seq and scRNA-Seq data using the same cut-offs: fold change > 1.5 (calculated in pseudobulk expression for scRNA-Seq) and adjusted *P* values (*P*_adj_) < 0.05.

### 2.13 Expression of *Itih4* in atherosclerosis using single-nucleus ATAC-Seq and scRNA-Seq analysis

We utilized previously published single-nucleus ATAC-seq to investigate the accessible chromatin patterns around the *ITIH4* gene in human atherosclerotic plaque cells.^26^ The data contain five major cell types: ECs, SMCs, macrophages, natural killer/T cells, and B cells. To further investigate the role of *Itih4* in cardiovascular disease, we also queried previously published scRNA-Seq of dedifferentiated SMCs from the thoracic aorta of mice with atherosclerotic disease (Ldlr^−/−^, ApoB^100/100^).^25^

### 2.14 The colocalization analysis for variant rs77347777 with existing genome-wide association study data

The coronary artery disease (CAD) SNP in *ITIH4* gene, rs77347777, was used for colocalization analysis between existing CAD genome-wide association study (GWAS) data,^27^*ITIH4* GTEx splice quantitative trait loci (QTL) (sQTL) data (aorta) (GTEx Consortium, 2020), and ITIH4 blood protein measurements.^28^ The National Institutes of Health (NIH) tool ezQTL^29^ was used to test colocalization analysis between GWAS and QTL data with 1000 Genomes LD of all populations and a cis-QTL distance of +/−75 kb.

### 2.15 Identification of splicing junctions leading to exon exclusion in *ITIH4*

The GTEx.V8 blood vessel data set was used to analyse splicing junctions in the aorta, coronary artery, and tibial artery using the recount3 and dasper R packages. The recount3 package create_rse() function^30^ was used to download junction reads as a SummarizedExperiment object. The dasper package’s junction_process() function^31^ was then used to annotate, filter, and normalize the junctions. The GenomicState R package (http://research.libd.org/GenomicState/) was used to load hg38 reference annotation as a TxDb object. Junctions with a count below five were filtered out, and normalization was performed by dividing the number of reads for each junction (that shares acceptor or donor site) with the total number of reads in its cluster. The rs77347777 SNP GTEx genotype information was used to divide the samples into CC, CT, and TT genotype groups, and the average of the normalized junctions was calculated to visualize the percentage of exon skipping with respect to genotype. All GTEx v8 individuals with genotype data and RNA-Seq data for aorta, coronary artery, or tibial artery were included. *ITIH4* exons were numbered according to the GRCh38 Ensembl release 108 canonical transcripts for ITIH4 (ENST00000266041.9). RNA-Seq exon–exon junction read counts for each individual were obtained from Recount3.^30^ The Recount3 junctions used were chr3:52814072-52816883 (exon21-exon23), chr3:52814364-52816883 (exon21-exon22), and chr3:52814072-52814208 (exon22-exon23). Finally, the plot_sashimi() function was used to inspect the unannotated splicing junction of interest near the rs77347777 SNP.

### 2.16 Correlation analysis of ITIH4 with atherosclerosis phenotype in cellular models

To assess its functionality, we utilized *in vitro* SMC expression and phenotype data collected from 151 multiethnic heart transplant donors.^32,33^ In particular, correlation analyses were conducted between the expression of *ITIH4* in SMCs and atherosclerosis-relevant phenotypes such as calcification.

### 2.17 Correlation and coexpression network analyses

The gene expression data from SMC^TRAP^ and SMC^TRAP-AS^ samples were preprocessed by filtering out genes with low expression by applying a minimum count of 5 and a total count of 15, resulting in a data set of 13 670 genes. VST transformation (DESeq2 package) was applied, and weighted gene coexpression network analysis (WGCNA)^34^ was conducted with a soft power of 14, determined through pickSoftThreshold(), and gene modules were identified using blockwiseModules() with a signed TOM. The *Itih4*-containing gene module was subjected to GO and STRING analysis. Differential correlation analysis was conducted for the experimental conditions (control and atherosclerotic mice). This analysis focused on gene pairs located three hops away from the *Itih4* gene, with data sourced from STRINGdb.

### 2.18 Spatial transcriptomics of human atherosclerosis tissue sections

Atherosclerotic plaques were obtained from patients undergoing carotid or femoral endarterectomy procedure at Kuopio University Hospital, Kuopio, Finland. The study was approved by the local Research Ethics Committee of the Northern Savo Hospital District (Decision No 139/2015). The study was performed in accordance with the declaration of Helsinki, and all patients gave written informed consent before the study started.

Freshly dissected human vascular samples were frozen in 2-methylbutane (Sigma-Aldrich 270342) and sectioned with a cryostat (Leica 3050S), and 10-µm-thick sections from one plane were placed within the capture areas of cold Resolve BioSciences slides. Molecular Cartography in situ hybridization (ISH)-based spatial transcriptomics platform (Resolve BioSciences) was performed as a service by the platform manufacturer. The final image from Resolve was performed in ImageJ^35^ using the Polylux tool plugin from Resolve BioSciences to examine specific Molecular Cartography signals.

### 2.19 Statistical analysis

For normally distributed samples, we performed an unpaired two-tailed *t*-test to evaluate the statistical significance of the mean difference between the groups. In cases where the data did not follow a normal distribution, we utilized the Mann–Whitney test to compare the two groups. The type of test used for each comparison is indicated in the figure legends. All statistical analyses were carried out using R version 4.0.2. *P* value < 0.05 was considered statistically significant [for RNA-Seq, false discovery rate (FDR)-adjusted *P* value < 0.05].

## 3. Results

### 3.1 Generation of αSMA-EGFP-L10a (SMC^TRAP^) transgenic mouse line to study SMC-specific gene expression in atherosclerosis

**Figure 1:**
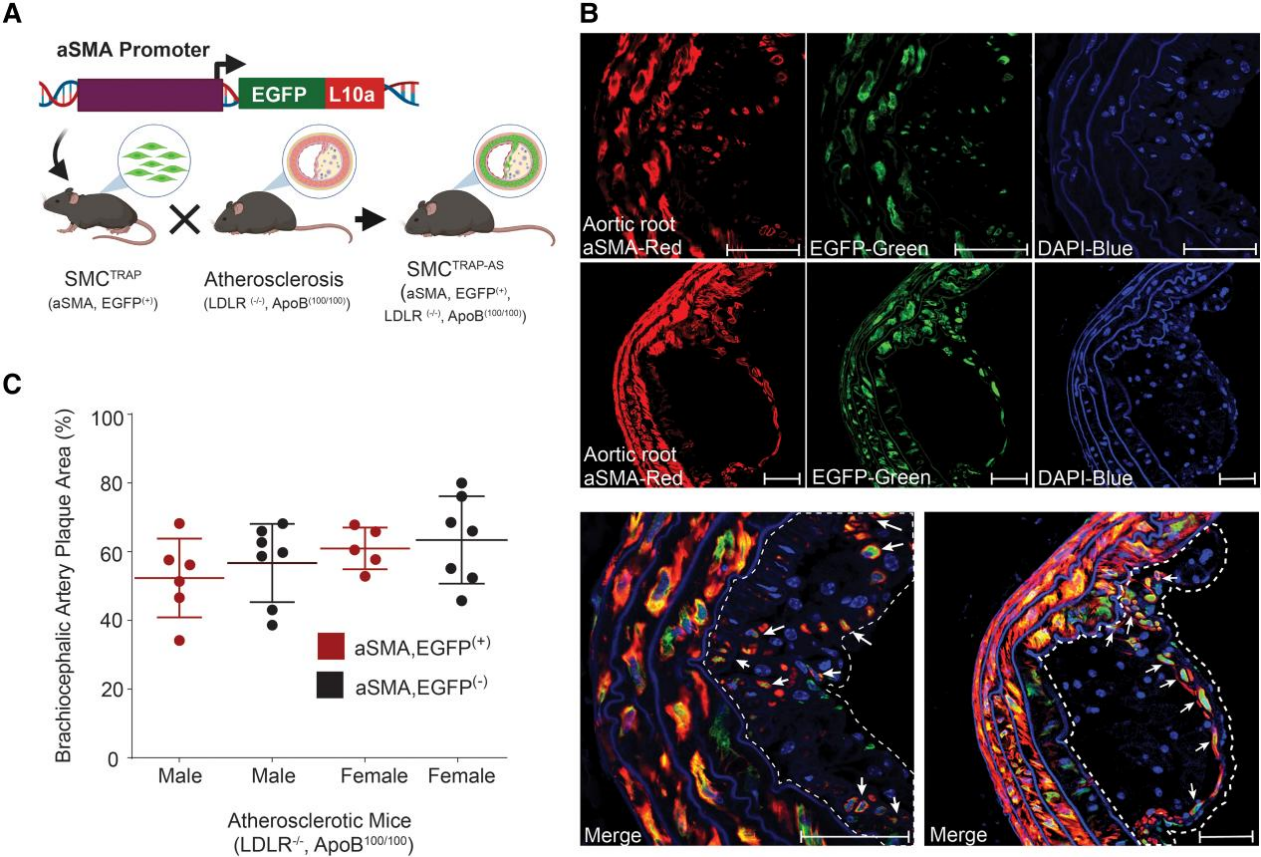
Development of transgenic mice for TRAP analysis of SMCs. (*A*) Construction of transgenic mice with expression of EGFP-tagged ribosomal protein L10a specifically in SMCs (SMC^TRAP^) and subsequent crossing with atherosclerotic mice (Ldlr^−/−^, ApoB^100/100^; Athero) to obtain SMC^TRAP-AS^ mice. Illustration created with BioRender. (*B*) Immunofluorescence microscopy of atherosclerotic lesions from SMC^TRAP-AS^ mice. Aortic sections show anti-αSMA staining using Cy3 (red/left subpanels), anti-EGFP using Alexa Fluor 488 (green/middle subpanels), and DAPI (blue/right subpanels). The imaging channels are presented individually (upper panels) and merged (bottom panels). The dashed lines indicate the atherosclerotic plaque, and the arrows highlight cells in the plaque interior and cap regions that are costaining for EGFP and αSMA. The images are representative of results from *n* = 3 mice. Scale bar: 50 µm. (*C*) Atherosclerotic plaque area in brachiocephalic artery sections from EGFP-tagged (EGFP^(+)^; *n* = 6 males and 5 females) and non–EGFP-tagged (EGFP^(−)^, *n* = 7 males and 7 females) mice. The plaque area relative to the lumen area (derived from the medial layer perimeter) is shown as mean ± S.D.

We generated a transgenic mouse model expressing an EGFP-ribosomal protein L10a fusion protein (EGFP-L10a)^18^ under the control of the SMC actin (αSMA; *Acta2* gene) promoter^36,37^ and crossed it with Lldr^−/−^, ApoB^100/100^ (Athero) mice^21,38^ (*Figure 1A*) to investigate SMC-specific gene expression changes during atherosclerosis. αSMA was chosen as the promoter because is a highly expressed marker for contractile SMCs, yet it also persists in the fibrous cap^39,40^ and exhibits sustained promoter activity, albeit at reduced levels, in SMCs exhibiting a synthetic phenotype.^41^ Immunofluorescence staining revealed colocalization of EGFP-L10a fluorescence with SMC (detected using anti-αSMA antibody) in the aorta, including in cells located in the interior of the plaque and in the fibrous cap (*Figure 1B*). Using EGFP fluorescence microscopy, EGFP-positive cell populations were evident in the aorta (medial layer, plaque interior, and cap), kidney, liver, and small intestine (see Supplementary material online, *Figure S1A*). To assess the potential impact of the SMC^TRAP^ transgene on atherosclerosis disease parameters, we analysed data from 4-month-old HFD mice. Blood plasma biochemistry parameters revealed that there were no significant differences in HDL cholesterol and TGs between SMC^TRAP-AS^ and EGFP-negative atherosclerotic mice (see Supplementary material online, *Figure S1B*). In male mice, the introduction of the EGFP-L10a transgene led to statistically significantly reduced levels of TC, LDL cholesterol, and body weight (see Supplementary material online, *Figure S1B*). Nevertheless, EGFP-positive (SMC^TRAP-AS^) and EGFP-negative mice demonstrated comparable plaque area (*Figure 1C*) and morphology (see Supplementary material online, *Figure S1C* and *D*) in brachiocephalic arteries in both males and females. Thus, the TRAP transgene construct does not appear to interfere significantly with plaque development in this atherosclerosis mouse model.

### 3.2 Capturing SMC-specific mRNA expression in atherosclerosis using high-throughput RNA sequencing

**Figure 2:**
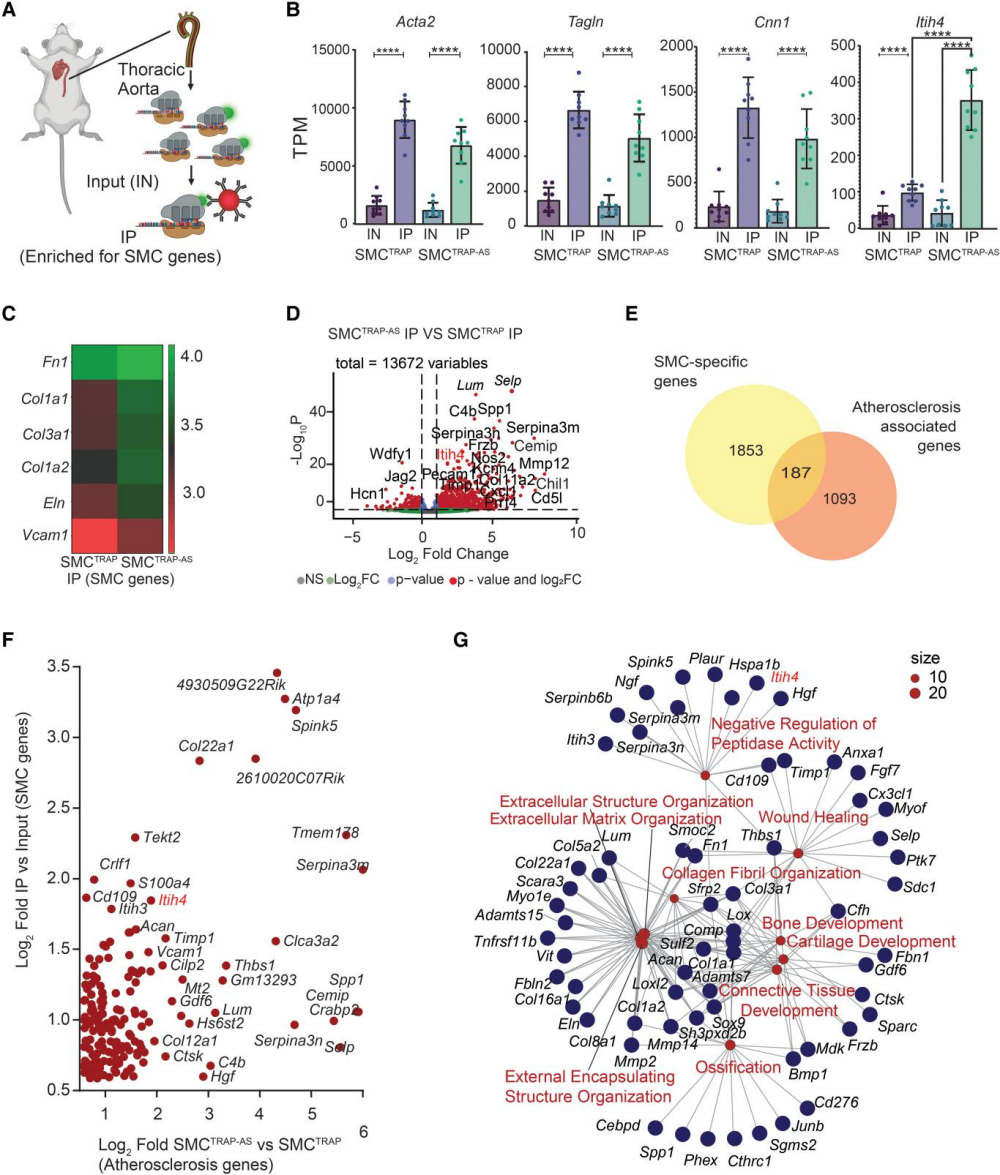
Sequencing of TRAP-Seq RNA and total RNA (*n* = 9 mice per genotype). (*A*) Schematic overview of the experiment. EGFP-tagged at ribosomal protein L10a is captured using anti-EGFP antibody to obtain an RNA fraction enriched with SMC genes (IP). Before antibody capturing, 5% of lysate is separated to be used as unenriched Input (IN). (*B*) SMC cell type marker gene (*Acta2*, *Tagln*, and *Cnn1*) and *Itih4* RNA enrichment in IP. Figures show the mean ± S.D. The two-tailed *t*-test was used to assess statistical significance (*P < 0.05, **P < 0.005). (*C*) Heatmap known synthetic SMC markers in IP RNA fraction of SMC^TRAP^ and SMC^TRAP-AS^ mice. Gene expression is presented as of log10 of average CPM. (*D*) DE analysis results comparing the IP fraction of atherosclerotic and non-atherosclerotic mice. (*E*) Intersection of genes defined as atherosclerosis-upregulated (SMC^TRAP-AS^ vs. SMC^TRAP^) and SMC-enriched (IP vs. Input). (*F*) Scatter plot of SMC specificity (y-axis) and atherosclerosis specificity (x-axis). The 187 intersecting genes from panel *E* are included. The top right corner shows the genes that are most highly enriched in both comparisons, the right side shows genes that are highly upregulated in atherosclerosis, and the top shows the most SMC-enriched genes. (*G*) Functional annotation enrichment analysis for the 187 genes from panel *E*.

High-throughput RNA sequencing was performed on RNA samples retrieved by anti-EGFP immunoprecipitation (IP) from the whole thoracic aortas of 15-month-old SMC^TRAP-AS^ and SMC^TRAP^ mice on regular chow (*n* = 9 per genotype; three male, six female). The unenriched RNA (‘Input’, equivalent to tissue bulk RNA-Seq) from the same samples was also sequenced (*Figure 2A*).

By comparing mRNA abundance in the IP and Input profiles, we first sought to confirm that TRAP-Seq captures genes expected to be enriched in SMCs. As depicted in *Figure 2B*, known SMC markers *Acta2*, *Tagln2*, and *Cnn1* were enriched in the IP fraction (a novel SMC-specific gene, *Itih4*, is also shown). In contrast, genes associated with other aortic cell types, including macrophages (*Csf1r*), pan-immune cells (*Ptprc*), adipocytes (*Adipoq*), mesenchymal/stromal (*G0s2*), and ECs (*Pecam1*), were not observed in the SMC-enriched (IP) fraction (see Supplementary material online, *Figure S2A*). To validate sensitivity towards the known genes implicated in phenotypic switching of SMCs in atherosclerosis, we confirmed that markers of synthetic SMCs (*Fn1*, *Col1a1*, *Col3a1*, *Col1a2*, *Eln*, and *Vcam1*) were elevated in SMC^TRAP-AS^ compared with SMC^TRAP^ (*Figure 2C*). Conversely, we noted a significant decrease in expression of contractile and quiescent smooth muscle markers such as *Acta2*, *Myh11*, *Tagln*, *Cnn1*, *Cald1*, and *Smtn* in SMC^TRAP-AS^ mice compared with SMC^TRAP^ within the IP-enriched mRNA fraction (see Supplementary material online, *Figure S2B*).

To decide how to consider sex in our TRAP-Seq data processing, we initially carried out TRAP-Seq analysis with either male or female samples alone or with both sexes combined (see Supplementary material online, *Tables S2*–*S11*). For both finding disease-upregulated genes as well as for discovering SMC-enriched genes, the sex groups revealed a substantial overlap in the resulting genes (see Supplementary material online, *Figure S2C* and *Tables S2 and S3*). Moreover, the statistically more significant results from single-sex analyses were more likely to be also detected in the sex-combined analysis and vice versa (see Supplementary material online, *Figure S2D*). Comparing the per-gene DE statistic from male-only and female-only TRAP-Seq analyses revealed a strong correlation between sexes (see Supplementary material online, *Figure S2E*). Finally, directly comparing male and female samples for each setting (see Supplementary material online, *Tables S4–S7*) revealed only a few differentially expressed genes, such as the sex chromosome genes *Xist* and *Ddx3y* in Input samples (see Supplementary material online, *Table S6*). Thus, in subsequent analyses, we focused on results obtained with both sexes combined.

Differential gene expression analysis (summarized in Supplementary material online, *Table S2*) identified a total of 2040 genes that were significantly enriched in SMCs (IP vs. Input; Supplementary material online, *Figure S2F* and *Tables S8, S9, and S12*) and 1280 genes that were upregulated in atherosclerosis in SMC IP samples and at the bulk tissue level [IP Athero vs. IP non-Athero (*Figure 2D*); Input Athero vs. Input non-Athero (see Supplementary material online, *Figure S2F* and *Tables S10, S11, and S13*)]. The intersection of these gene sets resulted in 187 genes (*Figures 2E* and *F*, Supplementary material online, *Table S14*), representing strong candidates for disease-associated SMC-specific genes. Supporting this, the GO analysis of these 187 genes revealed significant enrichment for functions related to the ECM, extracellular structure, and collagen fibril organization, ossification, and connective tissue development (*Figure 2G*, Supplementary material online, *Tables S15 and 16*). The gene list included well-known atherosclerosis-associated genes *Serpina3*,^42^*Cemip*,^43^*Thbs1*,^44^*Lum*,^45^ and *Spp1*^46,47^ but also novel genes without previous association to SMC function in atherosclerosis, such as *Anxa4*, *Cd276*, *Itih4*, *Myof*, *Pcdh11x*, *Rab31*, *Serpinb6b*, *Slc35e4*, *Slc8a3*, and *Spink5* (see Supplementary material online, *Table S17*).

### 3.3 Improved detection of atherosclerosis-upregulated genes in TRAP-Seq compared with bulk RNA-Seq

**Figure 3:**
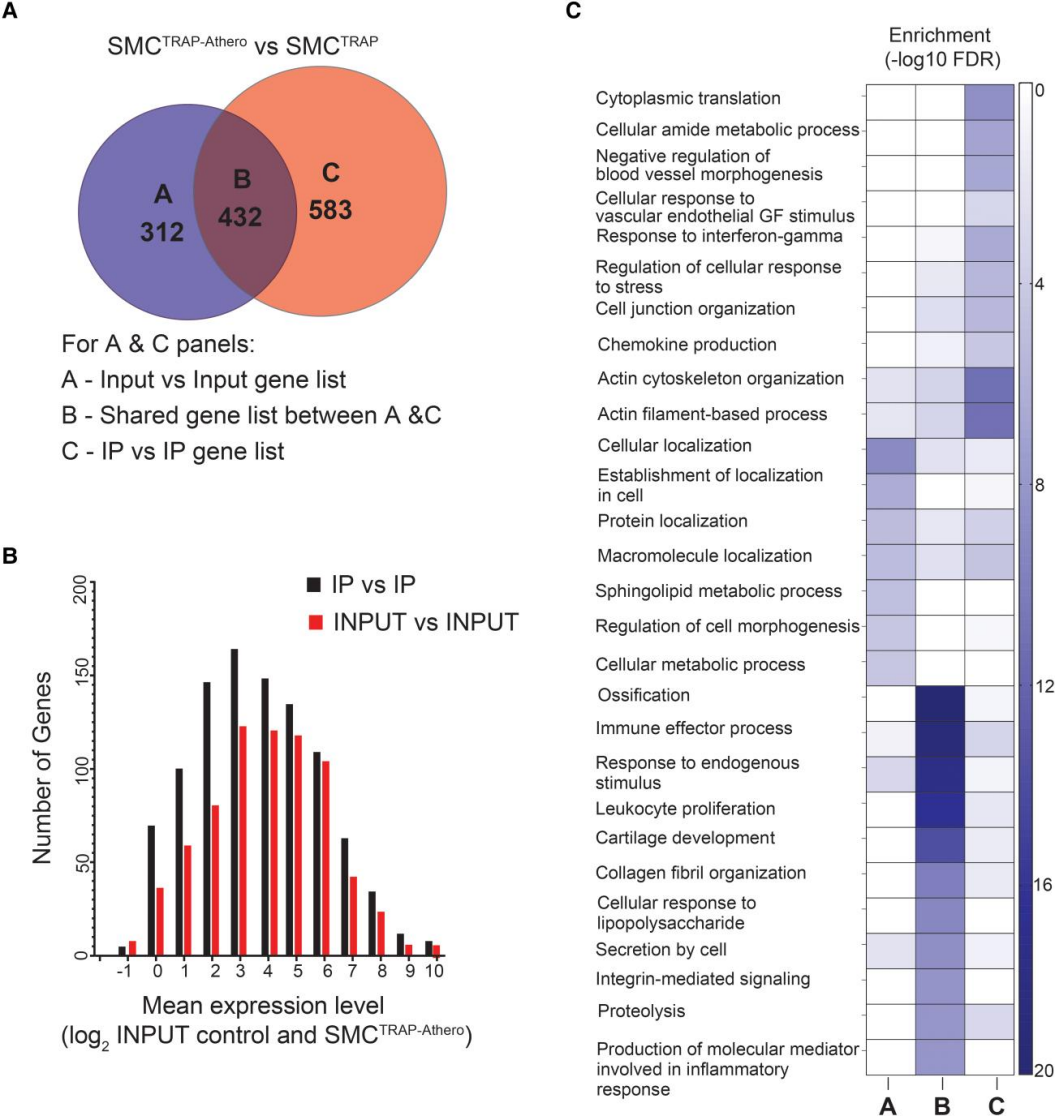
Comparison of disease-upregulated genes defined based on SMC-enriched RNA (IP) or total RNA (Input) samples (*n* = 9 mice per genotype). (*A*) Venn diagram showing the genes that are found as atherosclerosis-associated only in IP (583 genes), only in Input (312 genes), or found in both (432 genes). (*B*) Histogram of the bulk tissue expression level of disease-induced genes detected in the IP-enriched RNA sequencing or the Input RNA-Seq. The expression level in bulk tissue (unenriched RNA) is shown (mean expression across all Input libraries). (*C*) GO enrichment analysis of each gene list in panel *A*.

Subsequently, we examined the dissimilarities in the atherosclerosis-upregulated (Athero vs. non-Athero) gene sets, depending on whether the query was conducted on SMC-enriched (IP) or the bulk tissue (Input) RNA data. Applying an FDR cut-off of 0.05, we observed a slight increase of genes with statistical significance in the IP samples compared with the Input samples (*Figure 3A*). Additionally, the IP-based analysis uncovered a larger number of differentially expressed genes with lower average expression levels in the bulk tissue RNA (*Figure 3B* and Supplementary material online, *Tables S18 and S19*), indicating the SMC enrichment enabled the identification of diseases-associated genes that might have been missed in the bulk RNA analysis due to their low abundance in total tissue RNA. Notably, the IP-exclusive gene set (comprising 583 genes) displayed enrichment for vascular processes, while the shared gene set (comprising 432 genes) was enriched for leukocyte, cytokine, and ECM processes. Furthermore, the Input-exclusive gene set (comprising 312 genes) was enriched for processes related to nerves (*Figure 3C* and Supplementary material online, *Table S20*). These findings imply that the IP-based gene set comprises mRNAs with a distinct functional enrichment profile that is characterized by low expression levels in bulk tissue but can be detected using TRAP-Seq.

To study the sample size requirements for TRAP-Seq, we ran the DE analysis with multiple permutations of subsampling from *n* = 3 to *n* = 9 per group. The results revealed that for IP vs. Input (SMC-enriched genes), the number of significant results (FDR < 0.05; fold change > 1.5) tends to saturate around sample size (*n* = 5) (see Supplementary material online, *Figure S3A* and *Table S21*). For atherosclerosis vs. control in Input (disease-upregulated genes in bulk tissue RNA-Seq), using the same cut-offs, saturation is not yet reached at *n* = 9 (see Supplementary material online, *Figure S3A* and *Table S21*); thus, TRAP-Seq shows less sample size requirement compared with bulk RNA-Seq. To evaluate per-gene variation in the different steps of TRAP-Seq, we analysed the coefficient of variation in IP, Input, and the IP/Input ratio. As depicted in Supplementary material online, *Figure S3B*, median variation is lowest in IP fraction expression levels, slightly higher in Input, and further increases slightly in the IP/Input ratio. Analysing gene expression variability as a function of expression level revealed that the IP/Input calculation tends to reduce variability (compared with Input) for moderately/highly expressed genes but increases it among the lowly expressed genes [∼10 counts per million (CPM) in bulk/Input RNA; Supplementary material online, *Figure S3C*].

To compare the performance of TRAP-Seq and scRNA-Seq for detecting SMC-enriched genes, we utilized previously published scRNA-Seq from the same atherosclerosis mouse model (Ldlr^−/−^, ApoB^100/100^)^25^ and applied the same discovery cut-offs (FDR < 0.05 and fold change > 1.5, calculated from pseudobulk in the case of scRNA-Seq). Regarding sensitivity to detect SMC-enriched genes that are low abundance in bulk RNA, the methods displayed similar results, frequently nominating genes expressed at ∼10 transcripts per million (TPM) in bulk RNA (see Supplementary material online, *Figure S3D* and *Tables S22 and S23*). scRNA-Seq and TRAP-Seq detected a similar number of genes as SMC-enriched in control aorta; however, in disease, scRNA-Seq nominated more genes than TRAP-Seq (see Supplementary material online, *Figure S3D* and *Tables S22 and 23*). To assess the quality of these results, we used the Kyoto Encyclopedia of Genes and Genomes (KEGG) Vascular SMC Contraction gene set (mmu04270) as a positive control. Ranking all expressed genes by their calculated SMC specificity, we found that TRAP-Seq and scRNA-Seq both highly significantly recover this gene set, though scRNA-Seq performed slightly better (see Supplementary material online, *Figure S3E*).

Besides SMCs, vascular cells reported to express αSMA include ECs undergoing endothelial to mesenchymal transition (EndMT),^48^ pericytes, and fibroblasts. In microscopy, we did not detect EGFP signal in the endothelial lining (*Figure 1B*, Supplementary material online, *Figure S1A*). The TRAP-Seq data does not show enrichment of the hallmark EndMT transcription factors *Snai1* and *Snai2* or endothelial markers such as *Pecam1*, *Vwf*, and *Cdh5* (see Supplementary material online, *Tables S8* and *S9*). Using ∼100 markers of EndMT^48^ and 175 markers of arterial fibroblasts^4,26^ revealed only a minor fraction overlap with TRAP-Seq SMC-enriched genes (see Supplementary material online, *Figure S3F*). The pericyte marker *Rgs5* was significantly enriched in non-diseased samples (see Supplementary material online, *Table S8*), though it does not rank high by SMC specificity (position 1261) and does not reach significance in diseased samples (see Supplementary material online, *Table S9*), suggesting that minor contribution from pericytes could be occurring. Overall, the results support that the predominant signal detected in the TRAP-Seq likely originates from SMCs, at least in the current experimental conditions.

### 3.4 *Itih4* as a novel SMC gene associated with atherosclerotic diseases

**Figure 4:**
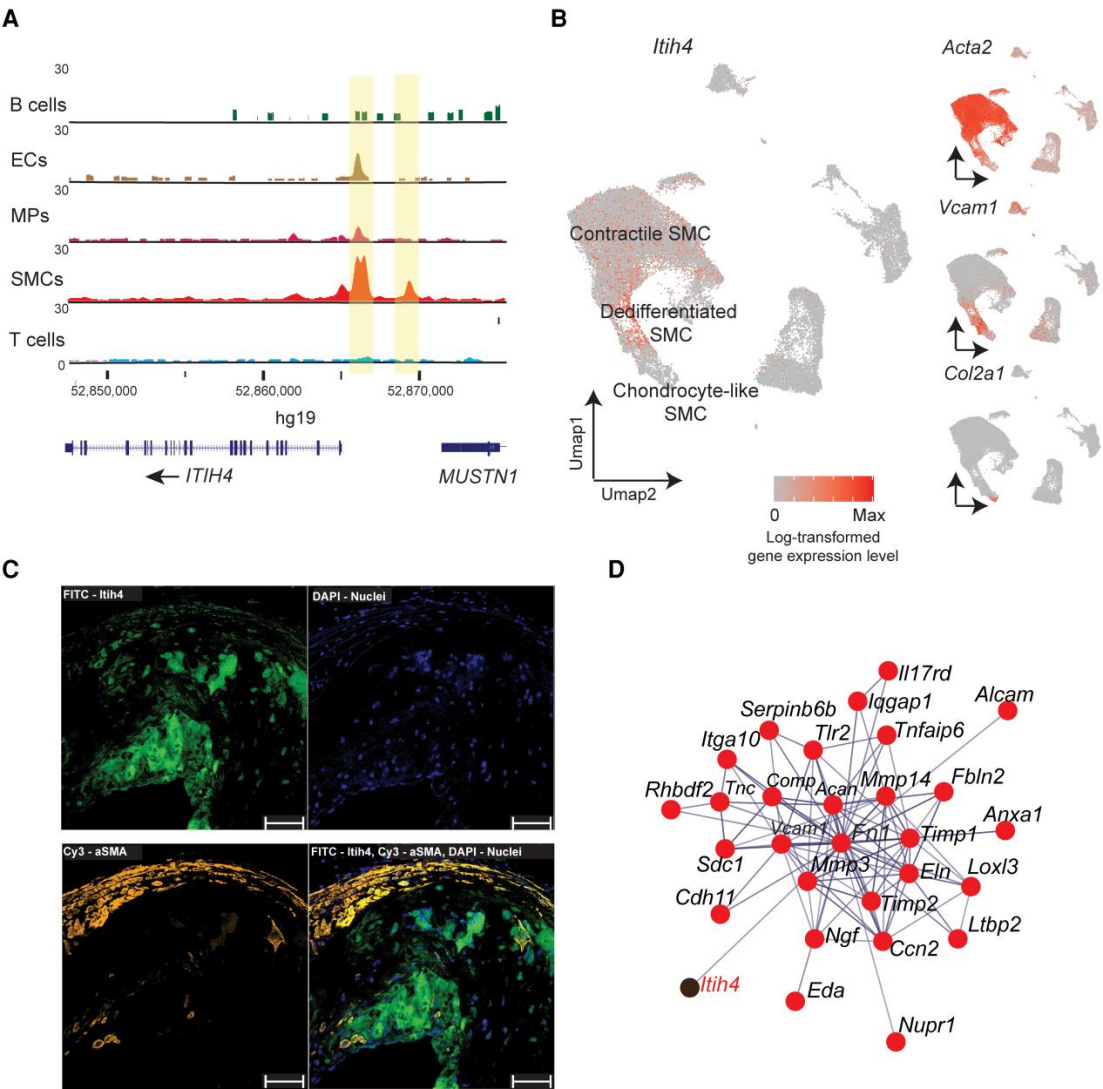
ITIH4 is expressed in atherosclerotic plaque SMCs. (*A*) Single-cell (sc)ATAC-Seq^26^ coverage tracks of the *ITIH4* gene region in human plaque cell types. ECs, endothelial cells; MPs, macrophages. The tracks are aggregated from three endarterectomy samples. (*B*) *Itih4* gene expression in scRNA-Seq^25^ of mouse atherosclerotic aorta. The cells are aggregated from four disease stages, each using three mice. (*C*) Immunohistological staining of anti-ITIH4 (FITC), anti-αSMA (Cy3), and nuclei (DAPI) in atherosclerotic plaque in mouse aortic root. A representative image is shown of *n* = 6 mice. Scale bar: 50 µm. (*D*) Coexpression network involving *Itih4* identified from atherosclerotic mouse TRAP-Seq data using WGCNA.

Our TRAP-Seq data from the atherosclerotic aorta revealed an enrichment of disease-specific SMC genes, as indicated by a higher number of genes with low expression in bulk RNA and specific GO terms. To further investigate this, we focused on *Itih4* gene, previously linked to liver inflammation and ECM remodelling.^49–51^ Human RNA expression data from GTEx and the Protein Atlas^52^ revealed high expression of *ITIH4* in the liver but additionally confirmed high expression levels in coronary and tibial arteries and aorta (see Supplementary material online, *Figure S3A*). ScATAC-Seq data analysis on human atherosclerosis^26^ revealed that the *ITIH4* gene promoter was highly accessible in SMCs (*Figure 4A*). We further scrutinized scRNA-Seq data^25^ from the thoracic aorta of atherosclerotic mice (Ldlr^−/−^, ApoB^100/100^) which confirmed that *Itih4* is most highly expressed in dedifferentiated (*Vcam1*+) SMCs in the aorta and is also expressed in contractile SMCs but is not significantly expressed in other cell types (*Figure 4B*). Immunohistochemistry in mouse revealed that ITIH4 sometimes, but not always, colocalizes with αSMA in atherosclerotic aortic root (*Figure 4C*), indicating that the plaque interior may contain cells that are αSMA-negative but ITIH4-positive at the protein level.

The *Iith4* gene is located in the same genomic region as *Itih1* and *Itih3*, while *Itih2* and *Itih5* are located near each other on a different chromosome. In our TRAP-Seq data, *Itih3* and *Itih2* are also SMC-enriched, but only *Itih4* enrichment increases with disease (see Supplementary material online, *Figure S4B*). Additionally, *Itih4* has the highest expression level of the family, ∼10-fold higher than the next highest member (see Supplementary material online, *Figure S4B*).

Gene coexpression network analysis of TRAP-Seq data (*Figure 4D*, Supplementary material online, *Figure S4C*) uncovered strongest coexpression for *Itih4* with matrix genes such as *Fn1* and *Vcam1*. The *Itih4*-containing gene module was related to but separate from the contractile smooth muscle gene module (*Acta2*, *Tagln*), suggesting functional differences (see Supplementary material online, *Figure S4C*). We subjected the *Itih4* module (288 genes; Supplementary material online, *Table S24*) to STRING protein function analysis, which revealed a subset the 29 genes (including *Itih4*) that are linked in terms of functionality as well as coexpression (*Figure 4D*, Supplementary material online, *Table S25*). These genes displayed enrichment for biological processes such as cell adhesion and ECM organization (see Supplementary material online, *Table S26*). We further performed a differential correlation analysis to compare *Itih4* coexpression in disease and control, which revealed a negative correlation with *Fn1* in healthy but a positive correlation in diseased aorta TRAP-Seq (see Supplementary material online, *Figure S4D* and *Table S27*). As an alternative approach, which is not informed by TRAP-Seq data, we queried the gene–gene functional interaction network for *Itih4* from GeneMANIA (https://genemania.org/). These results (see Supplementary material online, *Figure S4E*) associated *Itih4* with other *Itih* genes, cholesterol metabolism (*Abca1*, *Ldlr*), and complement (*Cfi*, *Hc*), a potential link to SMC proliferative phenotype.^53^

To study *ITIH4* expression in human atherosclerosis, we performed spatial transcriptomics in carotid and femoral endarterectomy samples using a panel of five genes (*ANPEP*, *COMP*, *FBLN1*, *IGFBP2*, and *ITIH4*), selected based on expression profile in human atherosclerosis scRNA-Seq and excluding highly expressed genes due to method limitations (risk of optical crowding interfering with the transcript decoding). The results revealed that *ITIH4* is expressed in a subset of *IGFBP2*+ cells, which is largely separate from the *IGFBP2*+*FBLN1*+ double-positive subset (see Supplementary material online, *Figure S5A*). *COMP*+*ANPEP*+ forms a further separate population with much lower *IGFBP2* expression (see Supplementary material online, *Figure S5A*). Each of these populations tended to concentrate in separate spatial regions rather than showing an intermixed spatial distribution (see Supplementary material online, *Figure S5A*). Based on human plaque scRNA-Seq cell populations^4^ (see Supplementary material online, *Figure S5B*) and their markers (see Supplementary material online, *Figures S5C* and *S6*), *IGFBP2*+*ITIH4*+ is characteristic of contractile and early transitioning SMCs, while *COMP+ANPEP*+ is characteristic of later transitioning SMC/fibromyocyte. *IGFBP2*+*FBLN1*+ is also descriptive of later transitioning SMCs (see Supplementary material online, *Figure S5C*). Thus, in human vessels, *ITIH4* mRNA appears to localize to unmodulated and early modulated SMCs.

### 3.5 Examination of *ITIH4* gene variant rs77347777 associated with cardiovascular disease risk

**Figure 5:**
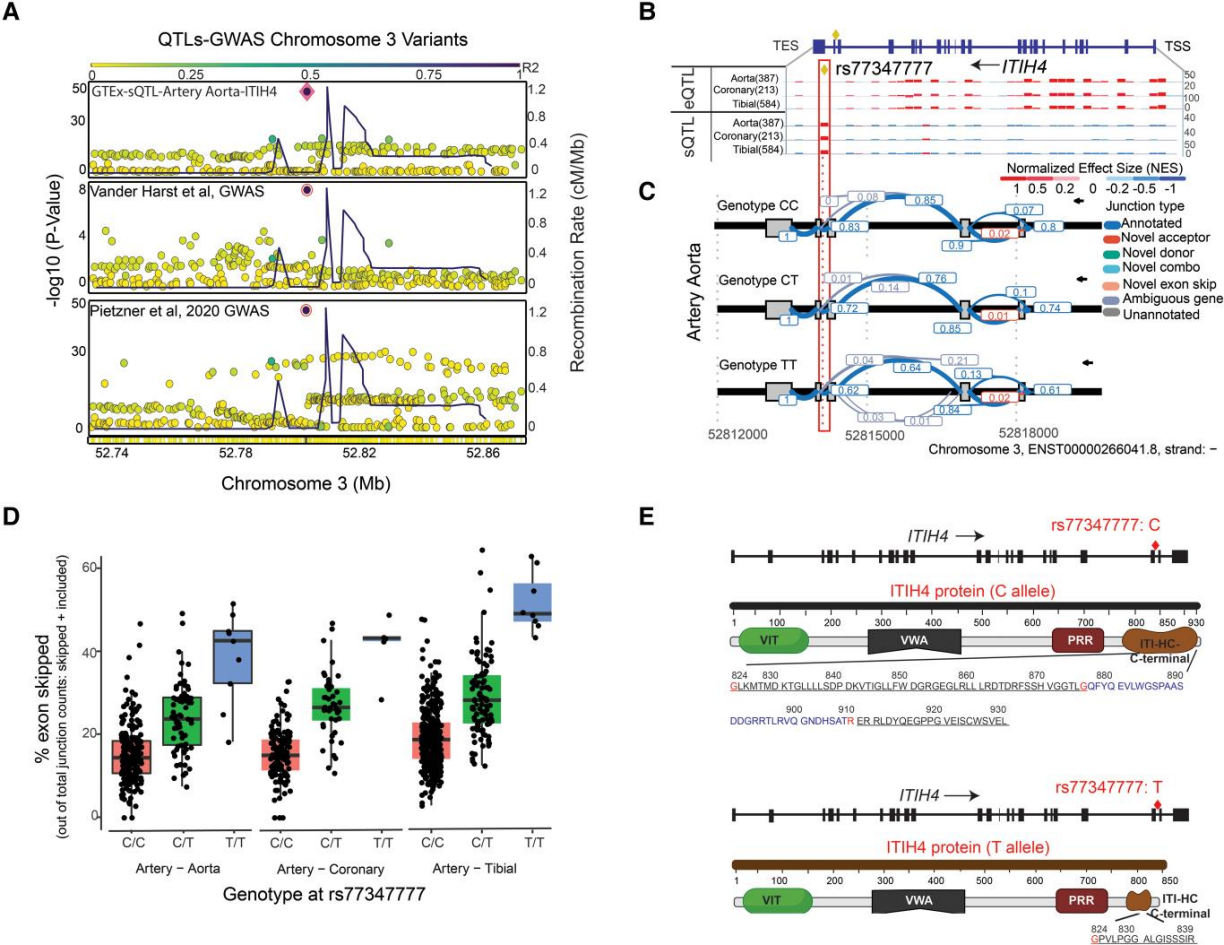
Colocalization of CAD GWAS and *ITIH4* molecular QTL signals at SNP rs77347777 located within the *ITIH4* gene. (*A*) Genetic colocalization of signals for of ITIH4 sQTL (GTEx aorta), CAD GWAS, and ITIH4 plasma protein QTL data. In the LocusZoom-style plots, the SNP rs77347777 is centered and highlighted with filled dot. (*B*) GTEx arterial tissue sQTL and expression (e)QTL results for *ITIH4* highlight rs77347777 as an sQTL but not eQTL. (*C*) Sashimi plot of exon junction usage in *ITIH4* mRNA near the rs77347777 SNP (location indicated with a diamond) in GTEx aorta samples. Samples were separated by genotype at rs77347777, and for each genotype, the average exon–exon junction usage is indicated. (*D*) Percentage of exon-skipped transcript by genotype in GTEx aorta, coronary artery, and tibial artery. The number of individuals with genotype C/C, C/T, and T/T is 225, 74, and 9 (aorta); 129, 44, and 5 (coronary artery); and 348, 98, and 8 (tibial artery), respectively. (*E*) Illustration of ITIH4 canonical protein and the exon 22 skipped form caused by rs77347777 showing the truncated ITIH4 protein with loss of C-terminal region.

Genetic associations for a particular gene can serve as strong evidence that the gene is involved in the underlying disease or trait. We therefore sought to investigate if such evidence exists for *ITIH4*. We identified several variants within the *ITIH4* gene that have been significantly associated with cardiovascular phenotypes, including heart rate (rs17331178, 3:52847544_C/A, *P*value 9.44e^−12^), pulse pressure (rs2071044, 3:52847601_C/T, *P* value 1.00e^−11^), and CAD (rs77347777, chr3:52848207:C:T, *P* value 8.86e^−11^) (Cardiovascular Disease Knowledge Portal; https://cvd.hugeamp.org/, March 2023) (see Supplementary material online, *Figure S7A* and *Table S28*). Of particular interest, the rs77347777 variant exhibited a significant association with CAD with *P* values of 1.51e^−9^ and 8.86e^−11^ (see Supplementary material online, *Figure S7B*),^27,54^ without other nearby variants in strong linkage disequilibrium (*Figure 5A*). This suggests that the observed association with CAD is likely due to rs77347777 variant and not to another variant that may be highly correlated with it. To confirm this, we used the ezQTL analysis tool from NIH (https://analysistools.cancer.gov/ezqtl/#/qtls), to conduct colocalization analysis between CAD GWAS and molecular QTL data. This analysis revealed strong colocalization between the CAD GWAS^27^ and *ITIH4* mRNA splicing QTL (GTEx) and plasma protein QTL^28^ (*Figure 5A*). The variant rs77347777 shows only sQTL effect but is not an eQTL variant (*Figure 5B*). Further analysis of the splice QTL data demonstrated that the rs77347777 strongly affects the relative abundance of an exon-skipped isoform of the *ITIH4* mRNA (*Figure 5C*). The percentage of exon skipping increases with the number of the CAD protective alternative allele T and can encompass a large fraction (up to 50%) of the total *ITIH4* mRNA pool in arterial tissues (*Figure 5D*).

The canonical *ITIH4* transcript (ENST00000266041.9) has 24 exons that translate into a 930 amino acid (aa) protein (see Supplementary material online, *Table S29*), and the exon skipping potentiated by the T allele of rs77347777 affects the 22nd exon (ENSE00003482020). Loss of exon 22 is expected to result in a frameshift after codon 824, which causes a premature termination codon (PTC) after 15 aa, and a total of 106 C-terminal aa are lost or replaced compared with the canonical protein, representing most of the C-terminal domain (*Figure 5E*, Supplementary material online, *Figure S7C* and *Table S30*). In the exon 22-skipped transcript, the PTC is located 48 nt upstream from the junction of exons 23–24, the last exon–exon junction of the transcript. This distance is shorter than the minimum of >50–55 nt thought to be required for nonsense-mediated RNA decay (NMD);^55^ thus, the transcript is expected to escape NMD, in line with the lack of an eQTL effect for rs77347777.

Finally, to evaluate the functional role of *ITIH4* in SMCs, we interrogated the published data on atherosclerosis-relevant cellular phenotypes studied across 151 human aortic SMC donors.^32,33^ In this data set, a negative correlation was observed between *ITIH4* expression level and SMC calcification (see Supplementary material online, *Figure S7D*). Further research is needed to confirm this association and to understand the specific mechanisms through which this variant may influence cardiovascular disease risk.

## 4. Discussion

Here, we developed transgenic mice with an EGFP tag on the ribosomal protein L10a, enabling efficient capture of SMC-specific mRNA in the context of atherosclerotic plaques using the TRAP-Seq method. Polysomal pulldown and affinity purification, followed by RNA sequencing, allowed for the simultaneous analysis of both cell type-specific genes and bulk mRNA transcripts in the same tissue sample with ∼10 times reduced cost compared with scRNA-Seq. By intersecting the SMC-enriched and atherosclerosis-enriched gene sets obtained from DE analysis, we were able to identify 187 genes that play a crucial role in ECM and connective tissue development, which have been found to be significantly enriched in SMCs associated with atherosclerotic disease. The identified genes in this study included a range of well-studied genes, each with their own distinct role in regulating the development of atherosclerosis. For instance, *Serpina3* has been shown to play a role in cell proliferation, migration, and expression of inflammation cytokines via NF-κB signalling pathways,^42^*Cemip* regulates the proliferation and migration of VSMCs in atherosclerosis through activation of the WNT-β-catenin signalling pathway,^43^*Thbs1* regulates plaque necrotic core formation and inflammation,^44^*Lum* contributes to collagen fibrillogenesis,^45^ and *Spp1* promotes the calcification in advanced stages of atherosclerosis.^46,47^ The SMC-enriched mRNA from TRAP-Seq (IP) showed enrichment for the expected smooth muscle and blood vessel–related biological processes. Interestingly, we also found functions related to axon and nervous system development, which could correspond to recent findings of Mohanta *et al.*^56^ who showed that atherosclerosis adventitia segments contain expanded axon networks, including growth cones at axon endings near immune cells and media SMCs.

In addition to our findings on known genes associated with SMC function and atherosclerosis, we also uncovered a set of novel genes, including *Itih4*. To our knowledge, the *Itih4* gene encodes for a secreted glycoprotein that has not been previously linked to SMC function or atherosclerosis. Our TRAP-Seq results were supported by histology and spatial transcriptomics analysis in both human and mouse tissue sections. Furthermore, previously published scATAC-Seq and scRNA-Seq data also supported *ITIH4* expression in plaque SMCs. Notably, two other *Itih* family members (*Itih3* and *Itih2*) were also found to be SMC-enriched in our data. While *Itih4* was the highest expressed member and the only one that had increased SMC-associated expression in disease, it is possible that the genes act in concert. Only ITIH4 was linked to splicing variants associated with CAD, leading to its selection for in-depth study; previous research has indicated ITIH3’s role in myocardial infarction.^57^ Additionally, ITIH3 has been recognized as a potential protein biomarker for CAD incidence in patients with familial hypercholesterolaemia.^58^ These findings suggest that further analysis of the ITIH family is warranted.

ITIH proteins are believed to play a role in maintaining the structure of the ECM, in addition to their role in plasma protease inhibition. Unlike other ITIH proteins, ITIH4 appears to lack the specific aa sequence required to bind to bikunin, a peptide that forms protease inhibitor complexes with other ITIH proteins.^59^ Previous studies have reported ITIH4 involvement in liver development and regeneration^49^ as well as its utility as an inflammatory indicator in diverse pathological conditions including bacterial infection,^60^ hepatitis C virus infection,^51^ recurrent pregnancy loss,^61^ and liver carcinoma.^50^ Studies have also reported ITIH4 as an anti-inflammatory marker in acute ischaemic stroke^62^ and Alzheimer’s disease.^63^ Our coexpression network analysis of atherosclerosis TRAP-Seq data linked *Itih4* to processes such as cell adhesion and ECM organization, correlated with genes such as *Fn1* and *Vcam1*.

We report here that *ITIH4* gene harbours an intriguing CAD GWAS hit, rs77347777, which is also splice QTL for *ITIH4* in human arterial tissues and a protein QTL for ITIH4 in the blood. The ITIH4 protein (930 aa; *Figure 5E* and Supplementary material online, *Figure S3E* and *Table S21*) is comprised of three major domains: vault protein inter-alpha-trypsin (VIT, PF08487, 36–146 aa), Von Willebrand factor type A (VWA) domain (PF00092, 274–455 aa), and inter-alpha-trypsin inhibitor heavy chain C-terminus (ITI-HC-C) domain (PF06668, 776–928 aa) (see Supplementary material online, *Figure S3E*). The VIT domain contains hydrophobic residues in N-terminal and aromatic aa at C-terminal region of its domain that aids in structural integrity of the domain, and any changes would affect the functionality of ITIHs.^64^ The VWA domain is evolutionarily conserved and well-studied for its involvement in cell adhesion. VWA aids in protein–protein interaction by forming the divalent cations using metal ion–dependent adhesion sites (MIDAS).^65^ The ITI-HC-C terminal domain represents the C-terminus of ITI-HCs; these heavy chains interact with hyaluronic acid that promotes the stability of ECM.^59^ Altogether, the domains of ITIH4 protein potentially serve two important functions. Firstly, similar to other ITIH proteins, they may facilitate ECM stabilization. Secondly, ITIH4 may act as a protease inhibitor using an unconventional mechanism.^66^ Beyond these functions, ITIH4 consists of a proline-rich region (PRR)^67^ and a tectonin-like β-propeller repeats.^68^ The ITIH4 cleavage in the PRR region/PSR (protease-susceptible region) region, which spans residues 633–713, has been linked to several human diseases, and it has been suggested that ITIH4 is a broad-acting inhibitor that targets numerous proteases.^66^ The canonical form of ITIH4 has 24 exons that codes for 930 aa, with 3 above-mentioned protein domains. The T allele of rs77347777 potentiates skipping of ITIH4 exon 22, leading to a frameshift after 824 aa and shortening protein from within the ITI-HC-C domain. The functional consequences of this remain unknown but based on the observed direction of association, it appears that the protective T allele, promoting exon 22 skipping, results in a pQTL effect in the blood.^28^ The pQTL association for rs77347777 was also seen by Ferkingstad *et al.*^69^ who additionally found that ITIH4 plasma protein level shows a positive correlation with coronary and peripheral artery disease (*P* value 4.80e^−27^ and 4.00e^−27^, respectively).

The utilization of the αSMA (*Acta2*) promoter in TRAP-Seq model allowed the detection of specific genes associated with SMCs, potentially leading to the identification of novel genes that have not been previously investigated. Alternative promoters could also be considered (e.g. *Myh11*, *Tagln*, or *Itga8*) and could have more specificity towards SMCs, as *Acta2* can also be expressed in EndMT ECs, pericytes, and myofibroblasts. Additionally, utilizing the *Acta2* promoter may result in an inability to detect gene expression from highly modulated SMCs that lack *Acta2* expression. As its benefits, *Acta2* tends to have a high mRNA expression level in SMCs and retains at least some expression in moderately dedifferentiated SMCs.^41^ The αSMA TRAP-Seq signal in our data appeared to predominantly originate from SMCs, both contractile and synthetic; however, in new experimental conditions, thought should be given towards other potential *Acta2*-expressing cell types.

Another limitation of our study is the lack of resolution to detect subtypes of SMCs. To address this, further studies could utilize existing single-cell RNA-Seq data from the same mouse model as a reference^25^ and apply computational deconvolution approaches^70^ for subtype detection in TRAP-Seq data. Additionally, we envision the SMC^TRAP-AS^ model being used to investigate disease-associated changes across other tissues relevant to disease aetiology. Furthermore, the use of TRAP technology in combination with RNase treatment enables the generation of ribosomal footprints that facilitate the tracking of ribosome-mediated codon-by-codon decoding activity. This approach could also serve as a powerful tool for identifying and characterizing conserved short open reading frames (sORFs) that may encode micropeptides relevant to the pathogenesis of atherosclerosis.

Altogether, our SMC^TRAP-AS^ model holds the potential to unveil novel genes that mediate the SMC specificity in the pathogenesis of atherosclerosis. The current evidence indicating the substantial involvement of SMCs in mediating the genetic risk of atherosclerotic cardiovascular disease^26,71,72^ supports integration of such knowledge with the rapidly evolving GWAS to discover prospective therapeutic targets. This approach can enable the discovery of candidate genes for drug development aimed at targeting alternative disease mechanisms, thereby complementing current treatment strategies.

**Translational perspective**

Atherosclerotic lesion development involves an interplay between multiple cell types, including immune, endothelial, and smooth muscle cells (SMCs). Despite this, there are no therapeutic options targeted specifically at vascular cells, possibly due to an incomplete understanding of cell type-specific alterations in disease. Here, we developed a murine model to specifically probe SMC transcriptional phenotype from tissue samples. This led us to uncover the SMC-specific expression of Itih4, a gene that harbours a CAD-associated genetic variant in humans. In the future, this mouse model could also be used to study other diseases with SMC involvement, such as gastrointestinal disorders.

## Supplementary Material

cvae028_Supplementary_Data

## Data Availability

The raw data from RNA-Seq experiments have been deposited in the NCBI GEO database (accession: GSE225424).
