# Supplementary material for: Translatome profiling reveals Itih4 as a novel smooth muscle cell–specific gene in atherosclerosis
Source: Cardiovasc Res. 2024 Jan 30;120(8):869–82. doi: 10.1093/cvr/cvae028 (PMC11218691; doi:10.1093/cvr/cvae028)
Supplement: cvae028_Supplementary_Data [file cvae028_supplementary_data.zip › Supplementary figure legends 2023-0362.docx]

**Translatome profiling reveals *Itih4* as a novel smooth muscle cell-specific gene in atherosclerosis**

Aarthi Ravindran^1^, Lari Holappa^1^*, Henri Niskanen^1^*, Ilya Skovorodkin^2^, Susanna Kaisto^2^, Mustafa Beter^1^, Miika Kiema^1^, Ilakya Selvarajan^1^, Valtteri Nurminen^1^, Einari Aavik^1^, Rédouane Aherrahrou^1,3^, Sanna Pasonen-Seppänen^4^, Vittorio Fortino^4^, Johanna P Laakkonen^1^, Seppo Ylä-Herttuala^1^, Seppo Vainio^2^, Tiit Örd^1‡**^, Minna U Kaikkonen^1‡**^

**AFFILIATIONS:**

1. A.I.Virtanen Institute for Molecular Sciences, University of Eastern Finland, Kuopio, 70211, Finland.
2. Biocenter Oulu and Faculty of Biochemistry and Molecular Medicine, University of Oulu.
3. Institute for Cardiogenetics, Universität zu Lübeck; DZHK (German Centre for Cardiovascular Research), Partner Site Hamburg/Kiel/Lübeck, Germany; University Heart Centre Lübeck, 23562 Lübeck, Germany.
4. Institute of Biomedicine, School of Medicine, Faculty of Health Sciences, University of Eastern Finland, 70211, Kuopio, Finland.

**‡Corresponding author: Tiit Örd** (**tiit.ord@uef.fi),** **Minna U Kaikkonen (minna.kaikkonen@uef.fi)**

***Equal second author contribution**

****Equal last author contribution**

**Keywords**

Smooth muscle cells; TRAP sequencing; Mouse models; atherosclerosis; *Itih4*; RPL10a

**Abbreviations**

EGFP – enhanced green fluorescent protein, ITI – Inter-alpha-trypsin inhibitor, SMC – Smooth Muscle Cell, TRAP – Translating Ribosome Affinity Purification.

**Supplementary Figure Legends**

**Supplementary figure 1. Characterization of a transgenic mouse model for translating ribosome affinity purification (TRAP) of smooth muscle (SMC) translated RNA.** (A) EGFP fluorescence imaging from fresh-frozen OCT-embedded tissues. Sections of aorta, liver, small intestine, and kidney are shown from SMC^TRAP-AS^ mice. EGFP is shown in green and DAPI in blue. The images are representative of results obtained from n=3 mice. Scale bar: 50 µm. (B) Body weight and blood plasma measurements for LDL cholesterol, HDL cholesterol, total cholesterol and triglycerides (TG) in EGFP-tagged mice (n=14 mice; 8 males and 6 females) and non-EGFP-tagged atherosclerotic mice (n=15 mice; 7 males and 8 females). Figures show the mean ± S.D. The two-tailed t-test was used to assess statistical significance (* p < 0.05, ** p < 0.005). (C-D) H&E staining of the brachiocephalic arteries of Ldlr^-/-^, ApoB^100/100^ atherosclerosis mouse model mice that either carried the EGFP TRAP transgene (C) or were EGFP-negative (D). In C and D, each section is from an individual mouse (n=12 mice per genotype) and the scale bar is 250 µm.

**Supplementary figure 2. Assessment of cell type specificity and sex effects in smooth muscle cell (SMC) TRAP-Seq.** (A) Markers of non-SMC cell types, such as immune cells (*Csf1r*, *Ptprc*), adipocytes (*Adipoq*), mesenchymal/stromal cells (*G0s2*) and endothelial cells (*Pecam1*) in the SMC-enriched (IP) and overall (Input) RNA samples. Figures show the mean ± S.D. The two-tailed t-test was used to assess statistical significance (* p < 0.05, ** p < 0.005). (B) Heatmap showing log10 of average CPM for known contractile/quiescent SMC markers in the IP-enriched RNA fraction of SMC^TRAP^ and SMC^TRAP-AS^ mouse aortas. (C-E) Comparison of TRAP-Seq analyses performed using only male (M; n=3), only female (F; n=6) or both sexes combined (n=9). Plots show the sharing of significant (FDR<0.05) differentially expressed (DE) genes (panel C), the fraction of shared DE genes as a function of statistical significance (panel D), and the correlation of per-gene differential expression statistic from DESeq2 runs using only males or only females (panel E). (F) Volcano plots illustrating DE analysis results with sexes combined. In panels A, B and F, n=9 mice per genotype. In panels C, D and E samples were separated by sex as indicated (n=3 for males; n=6 for females).

**Supplementary Figure 3. Analyses of TRAP-Seq sample size requirements, variation within groups, and comparison to single-cell RNA-Seq (scRNA-Seq) for defining smooth muscle cell (SMC)-enriched genes.** (A) Number of differentially expressed (DE) genes detected in TRAP-Seq as a function of sample size per group. DE genes were defined as FDR<0.05 and fold change >1.5 (i.e., SMC-enriched or disease-upregulated genes). Twenty permutations of subsampling were analyzed for each group size. (B) Coefficient of variation (CoV; calculated as S.D./mean) for all genes included in DE analysis. (C) Gene variability as a function of gene expression level in counts per million (CPM). (D) The bulk tissue expression level of genes detected as SMC-enriched by TRAP-Seq and scRNA-Seq. SMC-enriched genes were defined as >1.5-fold upregulated in SMC and FDR-adjusted P value < 0.05. (E) Enrichment for KEGG pathway mmu04270 (vascular SMC contraction; mouse) in genes ranked by SMC-specificity based on TRAP-Seq and scRNA-Seq. (F) Intersection of SMC-enriched genes defined based on TRAP-Seq with markers of previously published markers EndoMT or arterial fibroblasts. For panels B-F, the TRAP-Seq sample size was n=9 mice per genotype.

**Supplementary Figure 4. Tissue expression levels and co-expression patterns of *Itih4.*** (A) Expression of *ITIH4* gene in GTEx V8 tissues shows significant expression of *ITIH4* in the arteries, following liver tissue. (B) Expression levels of *Itih* family genes in mouse aorta TRAP-Seq (n = 9 mice per genotype) as transcripts per million (TPM). The mean ± S.D is shown. (C) Tree of co-expression modules from Weighted Gene Co-Expression Network Analysis (WGCNA) with modules containing *Itih* family genes highlighted. Additionally, the locations of *Csf1r* (macrophage marker), and *Acta2* and *Tagln* (contractile smooth muscle cell markers) are highlighted. (D) Differential correlation analysis comparing WGCNA networks generated from atherosclerotic and non-atherosclerotic TRAP-Seq IP samples. Genes connected to *Itih4* were analyzed. Cor_c1, correlation in SMC^TRAP-AS^ IP; Cor_c2, correlation in SMC^TRAP^ IP. (E) Gene-gene functional interaction network calculated for *Itih4* by GeneMANIA (<https://genemania.org/>).

**Supplementary Figure 5. Identification of *ITIH4* expression using spatial transcriptomics in human atherosclerotic lesions** (A) Molecular Cartography based identification of *ITIH*4 expression in human atherosclerotic lesions. Sections from one carotid and two femoral endarterectomy patients are shown. Each colored dot indicates a single-molecule transcript detection event. DAPI staining is shown in white. Scale bar: 100 μm. (B) Overview of previously published human atherosclerosis single-cell (sc)RNA-Seq data^4^ with major cell populations annotated. The cells are aggregated from 4 individuals. (C) Expression levels of the genes used in Molecular Cartography (*ANPEP*, *COMP*, *FBLN1*, *IGFBP2* and *ITIH4*) compared commonly used cell type markers based on scRNA-Seq cell populations shown in panel B.

**Supplementary Figure 6. Human atherosclerosis single-cell (sc)RNA-Seq gene expression plots of genes used in Molecular Cartography of human lesions (*ANPEP*, *COMP*, *FBLN1*, *IGFBP2* and *ITIH4*) and commonly used cell type markers.** scRNA-Seq cell populations shown in Figure S5B using data originally published by Wirka et al^4^. The cells are aggregated from 4 individuals.

**Supplementary Figure 7. Additional phenotypic traits associated with ITIH4.** (A) Variants near *ITIH4* that are associated with cardiovascular traits. The CVDKP portal (<https://cvd.hugeamp.org/>) listed significant genetic associations for heart rate (rs17331178), pulse pressure (rs2071044) and CAD (rs77347777). (B) LocusZoom plots for rs77347777 from two CAD GWAS studies. P values are 8.86e-11 and 1.51e-9 for Aragam et al., 2022 and van der Harst et al., 2017, respectively. (C) 3D model of the ITIH4 protein with its major domains VIT, VWA and ITI-HC-C terminus highlighted (SWISS-MODEL AF-Q14624-F1, <https://swissmodel.expasy.org/>). VIT, vault protein inter-alpha trypsin domain; VWA, Von Willebrand factor type A domain; ITI-HC-C, Inter-alpha-trypsin inhibitor heavy chain C-terminus domain. (D) Pearson correlation between ITIH4 expression and SMC calcification in a genetically diverse cohort of human SMC donors (n=151 individuals). *In vitro* calcification was carried out using the inorganic phosphate method.

**Supplementary Tables**

**Supplementary Table 1.** List of the mice strains and their genotype used in the TRAP-Seq and Immunohistology.

**Supplementary Table 2.** Summary of differential expression analysis in atherosclerosis and control groups across various RNA fractions using DESeq2 in individual male, female, and combined sex samples.

**Supplementary Table 3.** List of upregulated genes (P.adj < 0.05) in individual male, female, and combined sex samples across various RNA fractions (SMC^TRAP^ IP vs SMC^TRAP^ Input, SMC^TRAP-AS^ IP vs SMC^TRAP-AS^ Input, SMC^TRAP-AS^ IP vs SMC^TRAP^ IP and SMC^TRAP-AS^ Input vs SMC^TRAP^ Input), illustrated alongside a Venn diagram comparison.

**Supplementary Table 4.** Differential expression analysis for male (n=3) versus female (n=6) samples in individual RNA fraction SMC^TRAP^ Input using DESeq2.

**Supplementary Table 5.** Differential expression analysis for male (n=3) versus female (n=6) samples in individual RNA fraction SMC^TRAP^ IP using DESeq2.

**Supplementary Table 6.** Differential expression analysis for male (n=3) versus female (n=6) samples in individual RNA fraction SMC^TRAP-AS^ Input using DESeq2.

**Supplementary Table 7.** Differential expression analysis for male (n=3) versus female (n=6) samples in individual RNA fraction SMC^TRAP-AS^ IP using DESeq2.

**Supplementary Table 8:** Differential expression analysis for SMC^TRAP^ IP vs SMC^TRAP^ Input using DESeq2 with sexes combined (n=9 per group).

**Supplementary Table 9.** Differential expression analysis for SMC^TRAP-AS^ IP vs SMC^TRAP-AS^ Input using DESeq2 with sexes combined (n=9 per group).

**Supplementary Table 10.** Differential expression analysis for SMC^TRAP-AS^ IP vs SMC^TRAP^ IP using DESeq2 with sexes combined (n=9 per group).

**Supplementary Table 11.** Differential expression analysis for SMC^TRAP-AS^ Input vs SMC^TRAP^ Input using DESeq2 with sexes combined (n=9 per group).

**Supplementary Table 12.** SMC-specific genes: 2040 genes from differential expression of SMC^TRAP^- IP vs SMC^TRAP^-Input and SMC^TRAP-AS^-IP vs SMC^TRAP-AS^-Input with log_2_ fold change > 0.5 and with p.adj value < 0.05.

**Supplementary Table 13.** Atherosclerosis-specific genes: 1280 genes from the differential expression analysis of SMC^TRAP-AS^ IP vs SMC^TRAP^ IP and SMC^TRAP-AS^ Input vs SMC^TRAP^ Input with log_2_ Foldchange > 0.5 and with p.adj value < 0.05.

**Supplementary Table 14.** Identification of shared genes among SMC-specific and atherosclerosis-specific genes.

**Supplementary Table 15.** Gene Ontology (GO) enrichment study using gProfiler, for atherosclerosis-associated genes (1280), smooth muscle cell-specific genes (2040), and 187 common genes in atherosclerosis and smooth muscle cell genes using gProfiler (March 2023).

**Supplementary Table 16.** The results from the gene ontology analysis of biological processes (GO:BP) organized according to adjusted p-values (p < 1E-08) for each group, highlighting the top 10 GO:BP within each cluster.

**Supplementary Table 17.** Identification of understudied genes among the 187 genes prioritized for smooth muscle specificity and upregulation with atherosclerosis.

**Supplementary Table 18.** Mean expression level of genes in the Input (IN) fraction. Genes differentially expressed between SMC^TRAP-AS^ and SMC^TRAP^ are shown.

**Supplementary Table 19.** Mean bulk tissue expression distribution for genes upregulated in disease vs control (SMC^TRAP-AS^ vs SMC^TRAP^) in either the TRAP RNA fraction (IP vs IP) or in bulk RNA (Input vs Input).

**Supplementary Table 20.** Gene ontology studies using gProfiler (March 2023) for the unique genes in SMC^TRAP-AS^ IP vs SMC^TRAP^ IP (583), SMC^TRAP-AS^ Input vs SMC^TRAP^ Input (312) and shared genes (432).

**Supplementary Table 21.** Identification of experimental variation using DESeq2 with random subsampling to vary sample size (n=3 to n=9 per group). 20 permutations were analyzed per sample size.

**Supplementary Table 22.** Differential expression analysis between SMC and non-SMC cells in in control mouse aorta scRNA-Seq^25^. P values are from the Wilcoxon test.

**Supplementary Table 23.** Differential expression analysis between SMC and non-SMC cells in in atherosclerotic mouse aorta scRNA-Seq^25^. Cells from Ldlr^-/-^, ApoB^100/100^ mice fed high-fat diet for 3 months were used. P values are from the Wilcoxon test.

**Supplementary Table 24.** Gene modules identified by the Weighted Gene Co-Expression Network Analysis (WGCNA) at soft power 14 for gene expression data of SMC^TRAP^ and SMC^TRAP-AS^ samples.

**Supplementary Table 25.** STRING network analysis of a subset of 28 genes derived using multiple K-means clustering for the WGCNA *Itih4* (Purple) gene module.

**Supplementary Table 26.** Gene ontology analysis for subset of 28 genes using gProfiler (October 2023). Genes were selected using multiple K-means clustering for the WGCNA *Itih4* (Purple) gene module.

**Supplementary Table 27.** Differential correlation analysis performed on gene interactions located three hops away from the seed gene “*Itih4”* (STRINGdb), with samples from both control and atherosclerosis TRAP mice.

**Supplementary Table 28.** List of significant variants in ITIH4 gene for cardiovascular phenotype from cardiovascular disease knowledge portal (CVDKP) in March 2023. The datasets contributing to the integrative analysis to find significant variants for the following cardiovascular phenotypes - heart rate (HR), pulse pressure and coronary artery diseases (CAD). The text in red indicates the LD Ref variant for each phenotype and our SNP of interest is highlighted in yellow.

**Supplementary Table 29.** Summary of human *ITIH4* (ENSG00000055955) transcript isoforms from ENSEMBL (GRCh38.p13).

**Supplementary Table 30.** Summary of protein coding exons for the human canonical *ITIH4* transcript (ENST00000266041) and the exon 22 skipped truncated protein sequence.
