# Supplementary figures and images for: Translatome profiling reveals Itih4 as a novel smooth muscle cell–specific gene in atherosclerosis
Source: Cardiovasc Res. 2024 Jan 30;120(8):869–82. doi: 10.1093/cvr/cvae028 (PMC11218691; doi:10.1093/cvr/cvae028)

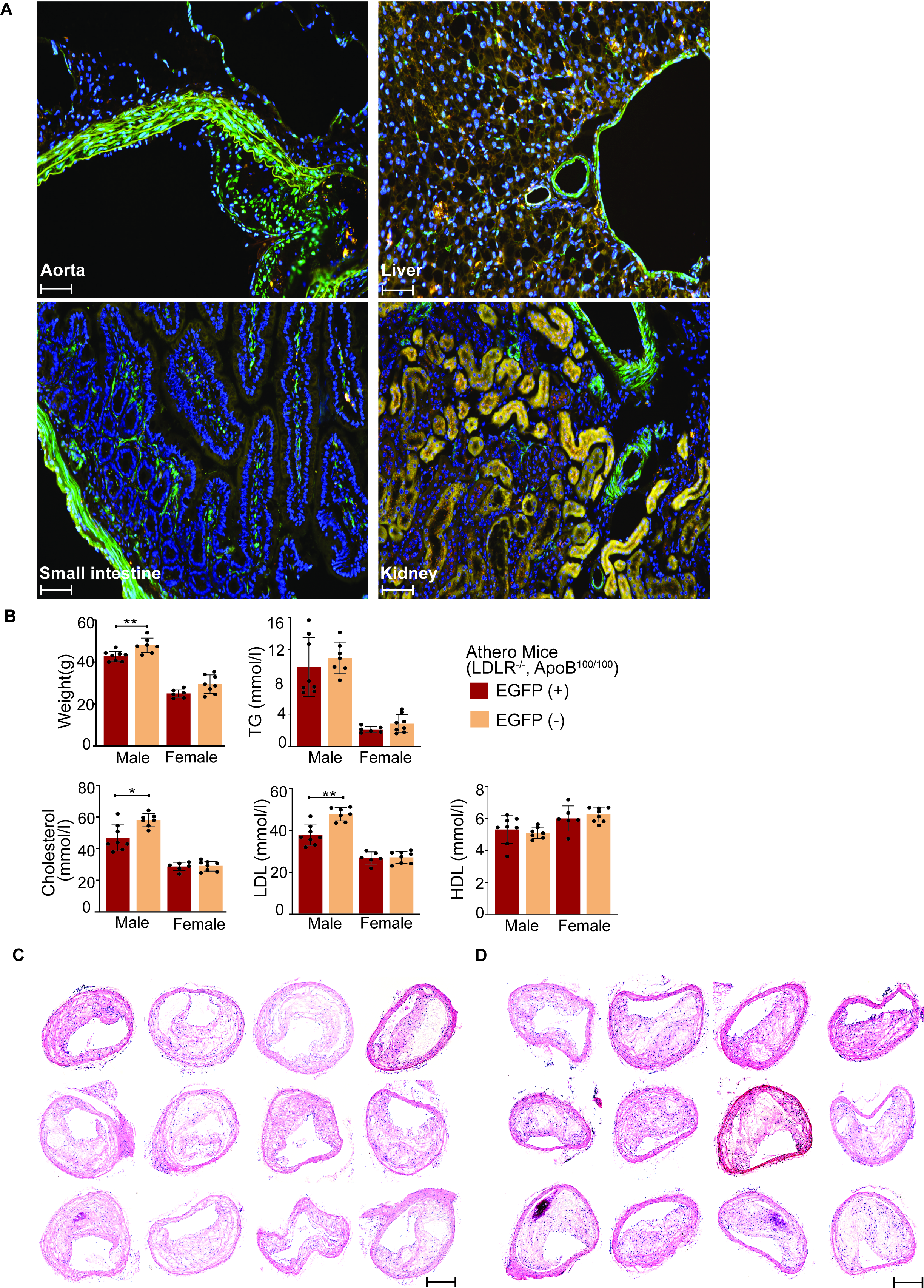

Supplement: cvae028_Supplementary_Data [file cvae028_supplementary_data.zip › Supp_Figure_1.tif]

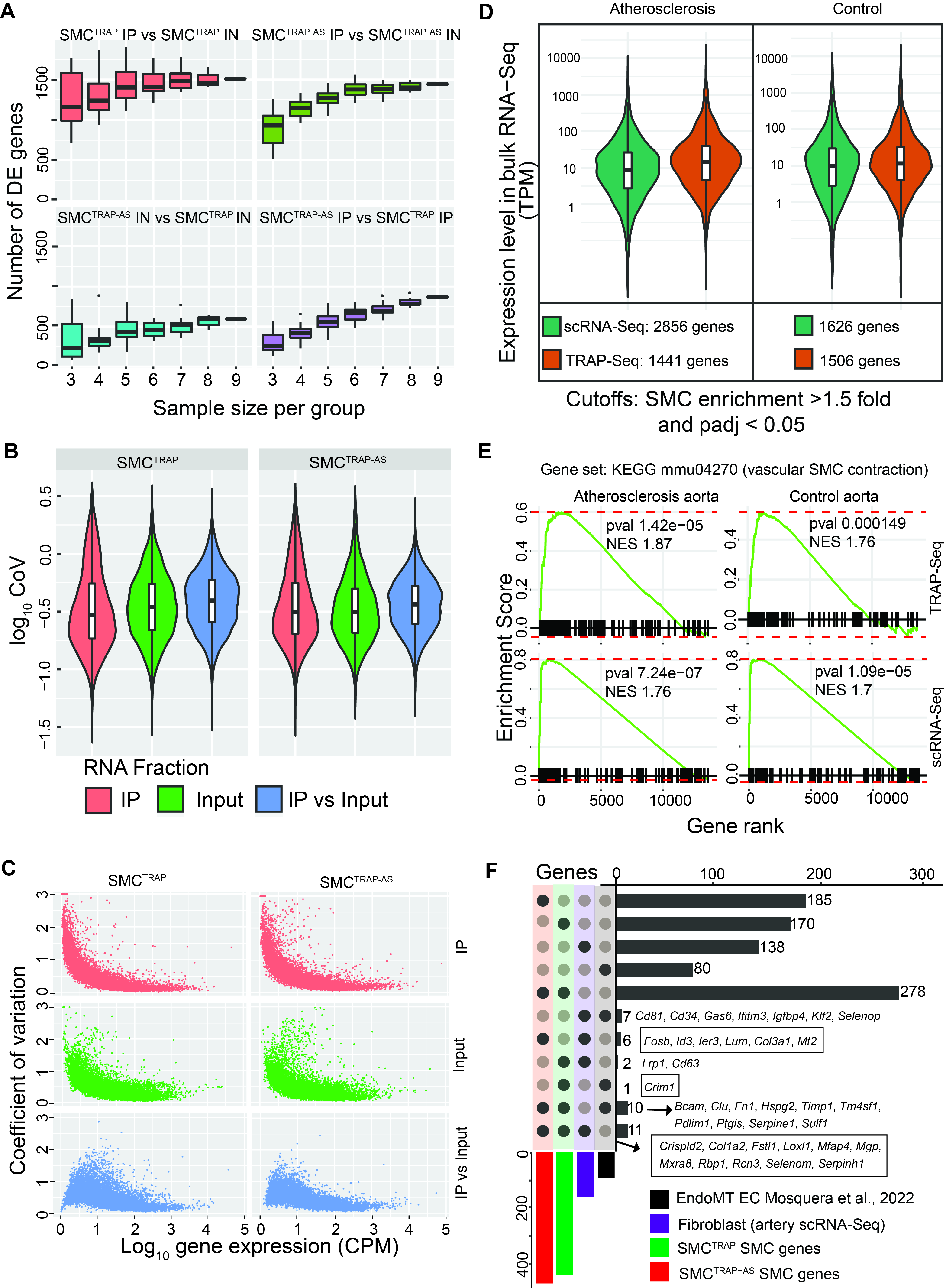

Supplement: cvae028_Supplementary_Data [file cvae028_supplementary_data.zip › Supp_figure_3.tif]

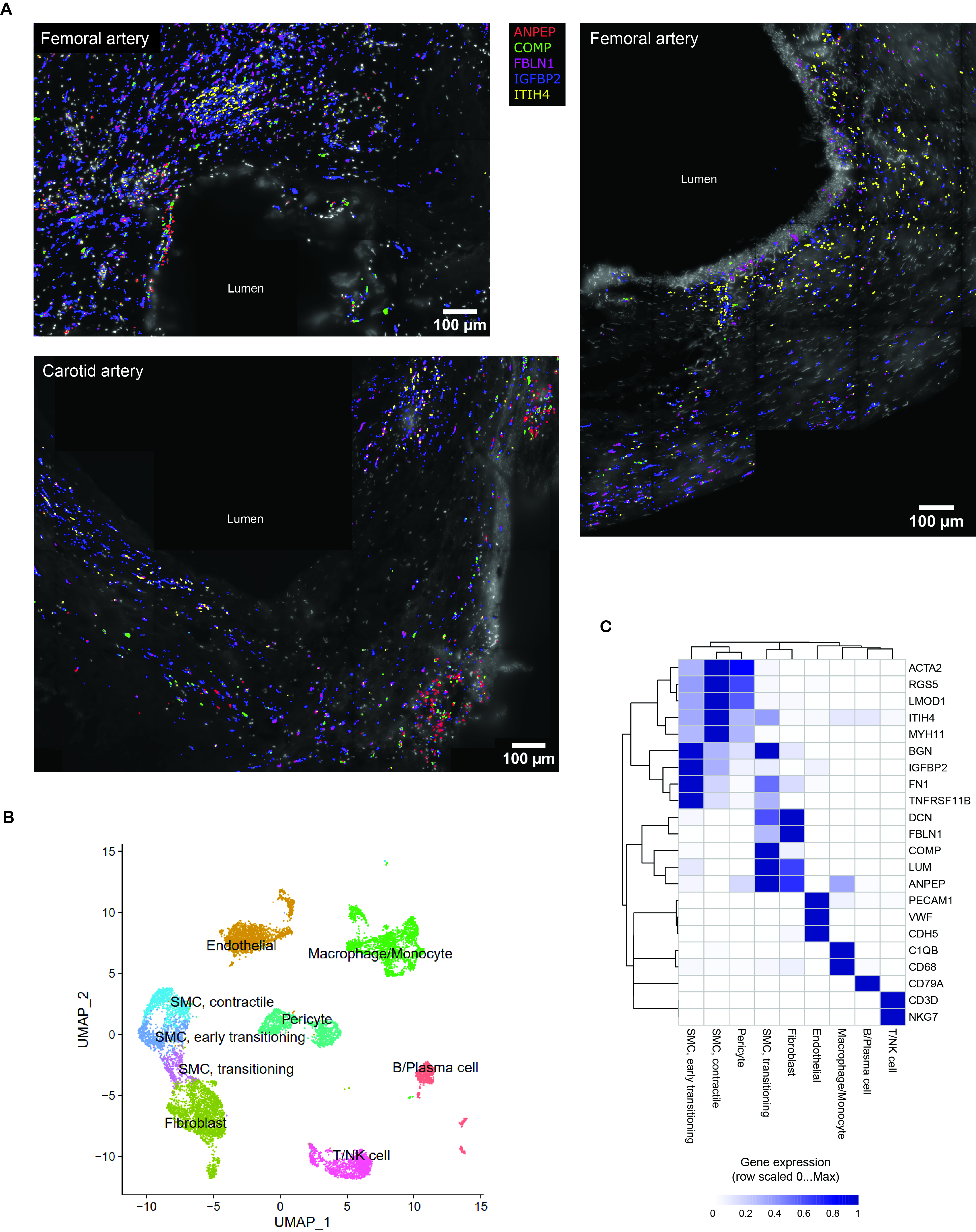

Supplement: cvae028_Supplementary_Data [file cvae028_supplementary_data.zip › Supp_Figure_5.tif]

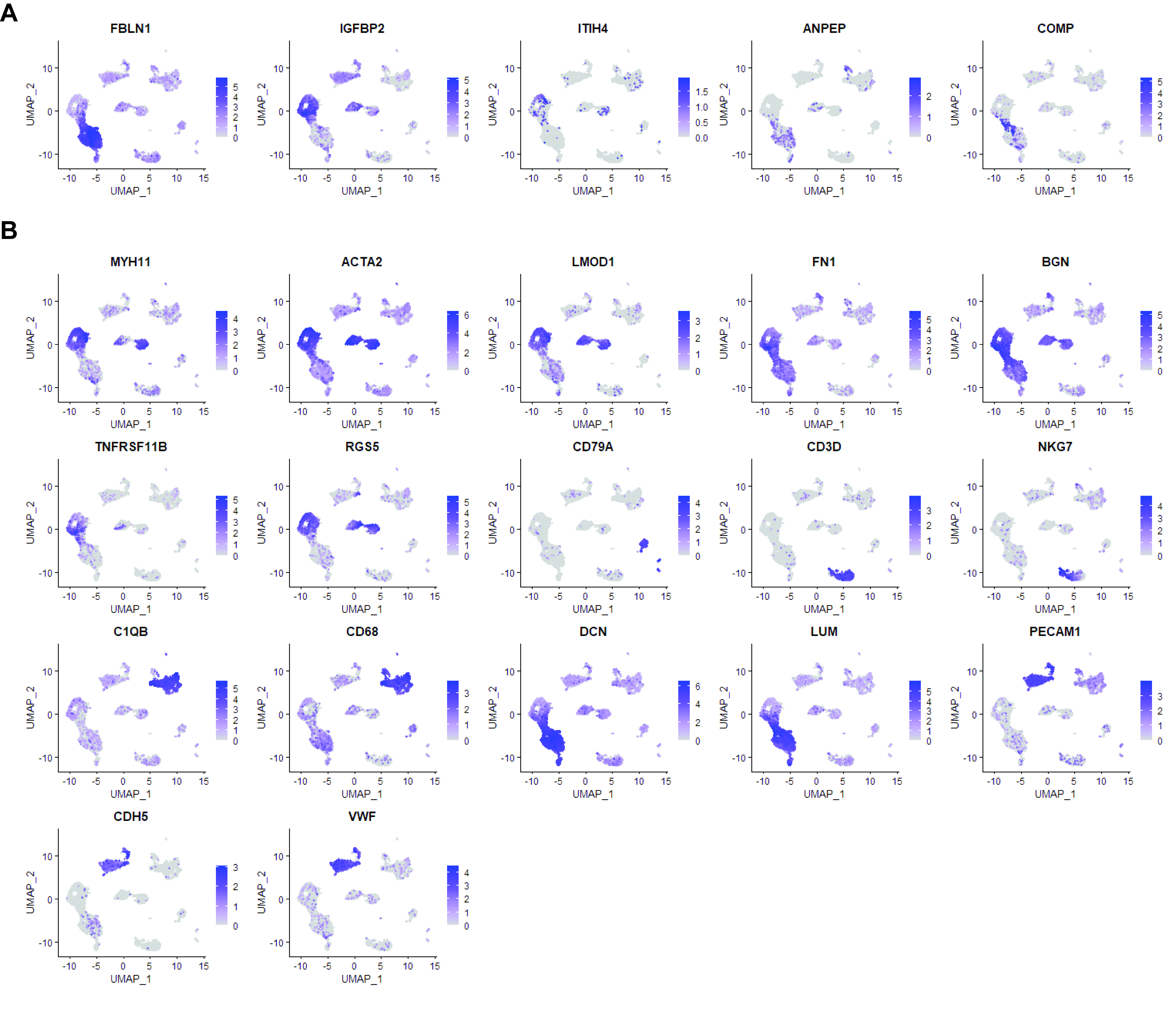

Supplement: cvae028_Supplementary_Data [file cvae028_supplementary_data.zip › Supp_figure_6.tif]
